# Oocyte exposure to ZnO nanoparticles inhibits early embryonic development through the γ-H2AX and NF-κB signaling pathways

**DOI:** 10.18632/oncotarget.17349

**Published:** 2017-04-21

**Authors:** Jing Liu, Yong Zhao, Wei Ge, Pengfei Zhang, Xinqi Liu, Weidong Zhang, Yanan Hao, Shuai Yu, Lan Li, Meiqiang Chu, Lingjiang Min, Hongfu Zhang, Wei Shen

**Affiliations:** ^1^ Key Laboratory of Animal Reproduction and Germplasm Enhancement in Universities of Shandong, Qingdao Agricultural University, Qingdao 266109, P. R. China; ^2^ Core Laboratories of Qingdao Agricultural University, Qingdao 266109, P. R. China; ^3^ State Key Laboratory of Animal Nutrition, Institute of Animal Sciences, Chinese Academy of Agricultural Sciences, Beijing 100193, P. R. China

**Keywords:** ZnO nanoparticles, oocyte stage exposure, embryo-toxicity, γ-H2AX, NF-κB

## Abstract

The impacts of zinc oxide nanoparticles on embryonic development following oocyte stage exposure are unknown and the underlying mechanisms are sparsely understood. In the current investigation, intact nanoparticles were detected in ovarian tissue *in vivo* and cultured cells *in vitro* under zinc oxide nanoparticles treatment. Zinc oxide nanoparticles exposure during the oocyte stage inhibited embryonic development. Notably, *in vitro* culture data closely matched *in vivo* embryonic data, in that the impairments caused by Zinc oxide nanoparticles treatment passed through cell generations; and both gamma-H2AX and NF-kappaB pathways were involved in zinc oxide nanoparticles caused embryo-toxicity. Copper oxide and silicon dioxide nanoparticles have been used to confirm that particles are important for the toxicity of zinc oxide nanoparticles. The toxic effects of zinc oxide nanoparticles emanate from both intact nanoparticles and Zn^2+^. Our investigation along with others suggests that zinc oxide nanoparticles are toxic to the female reproductive system [ovaries (oocytes)] and subsequently embryo-toxic and that precaution should be taken regarding human exposure to their everyday use.

## INTRODUCTION

Concerns over the embryo-toxicity of nanoparticles (NPs) have increased exponentially over recent years and numerous articles have been published [1–71]. Many animal models have been used to test NPs embryo-toxicity; these include zebrafish [2–8, 10, 11, 14–17, 20–22, 24–28, 31, 33, 35, 37, 38, 41, 43, 45–47, 49–51, 53–57, 59, 62, 64, 66–70], mice [1, 9, 12, 13, 42, 48, 52, 63, 71], rat [18, 19, 30, 32], chicken [36, 44], oryzias latipes [23, 29, 34, 58], Xenopus laevis [7, 60], sea urchin [40] Mytilus galloprovincialis (Lmk) [39], snail [61], and oyster [65]. Many different types of NPs have been tested using these animal models. The most popular NPs are silver (Ag) NPs [1, 3, 8, 12, 13, 16, 22–26, 28, 32, 33, 34, 37, 38, 41, 43, 48, 49, 55, 56, 65, 66, 70], then zinc oxide (ZnO) NPs [3, 7, 14, 15, 18–20, 26, 40, 47, 62], gold (Au) NPs [3, 29, 30, 45, 46, 50–52, 55, 66, 67], titanium dioxide (TiO2) NPs [9, 16, 17, 21, 25, 28, 31, 39, 63, 69], silicon dioxide (SiO2) NPs [3, 26, 35, 58, 63], Cd NPs [3, 26, 53, 61], chitosan NPs [4, 42, 57], quantum tots [11, 60, 68], Cu NPs [25, 27, 64], platinium (Pt) NPs [44, 55] and other NPs including CoFe2O4, selenium, diamond, Ni, F2O3, and lead NPs [2, 5, 6, 10, 26, 27, 36, 45, 54, 59, 69, 71]. Although many articles have been published exploring the embryo-toxicity of NPs, only their toxic effects on different embryos have been investigated and very few studies have examined the underlying mechanisms.

Austin *et al*. found that intravenous injection of Ag NPs (10 nm) into pregnant mice resulted in notable silver accumulation in the maternal liver, spleen, and visceral yolk sac, and might suppress embryonic growth [1]. Parivar *et al*. observed that TiO_2_ NPs caused significant changes in chondrocytes in the following developmental stages: resting, proliferating, hypertrophy, degenerating, perichondrium, and mesenchymal cells [9]. Park *et al*. investigated the effects of chitosan nanoparticles on mouse embryonic development, and indicated that they lowered the expression of both trophectoderm-associated genes and pluripotent marker genes. Yamashita *et al*. found that SiO_2_ and TiO_2_ nanoparticles caused pregnancy complications in mice with both types being found in the placenta, fetal liver, and fetal brain [63]. Hong *et al*. found significant reductions in fetal weights along with an increase in abnormalities after administration of ZnO NPs at a rate of 400 mg/kg/day; however, no significant difference was found in the Zn content of fetal tissue between the control and 400 mg/kg/day groups [18, 19]. Tsyganova *et al*. reported that Au NPs penetrated the rat placental barrier *in vivo*; however, no morphological changes took place in the liver, kidneys, spleen, and brain of fetuses [30]. Prasek *et al*. found that Pt NPs did not inhibit the growth and development of chicken embryos; however, it did induce apoptosis and cause a reduction in the number of proliferating cells in brain tissue [44]. In almost all published articles based on studies of nanoparticle embryo-toxicity, the embryos were tested directly (fertilized eggs of aquatic animals, or chickens) or indirectly (pregnant mouse or rat).

Although ZnO NPs are useful and novel material for a broad range of aspects, many reports have indicated that they may have adverse effects on organisms [72–77] and specifically on the reproductive systems [78, 79]. Nanoparticle safety, however, is not fully understood, particularly the effects and underlying mechanisms of ZnO NPs on embryonic development following oocyte stage exposure. Our results demonstrated that ZnO NPs inhibited embryo development during oocyte stage exposure, and that the γ-H2AX and NF-κB pathways might be crucial in this inhibition. Our investigation, along with others, suggests that ZnO NPs are toxic to female reproductive systems [ovaries (oocytes)] and are subsequently embryo-toxic. Precautions should therefore be taken *in situ*ations of human exposure.

## RESULTS

### Characterization of ZnO, CuO, and SiO2 NPs

**Figure 1:**
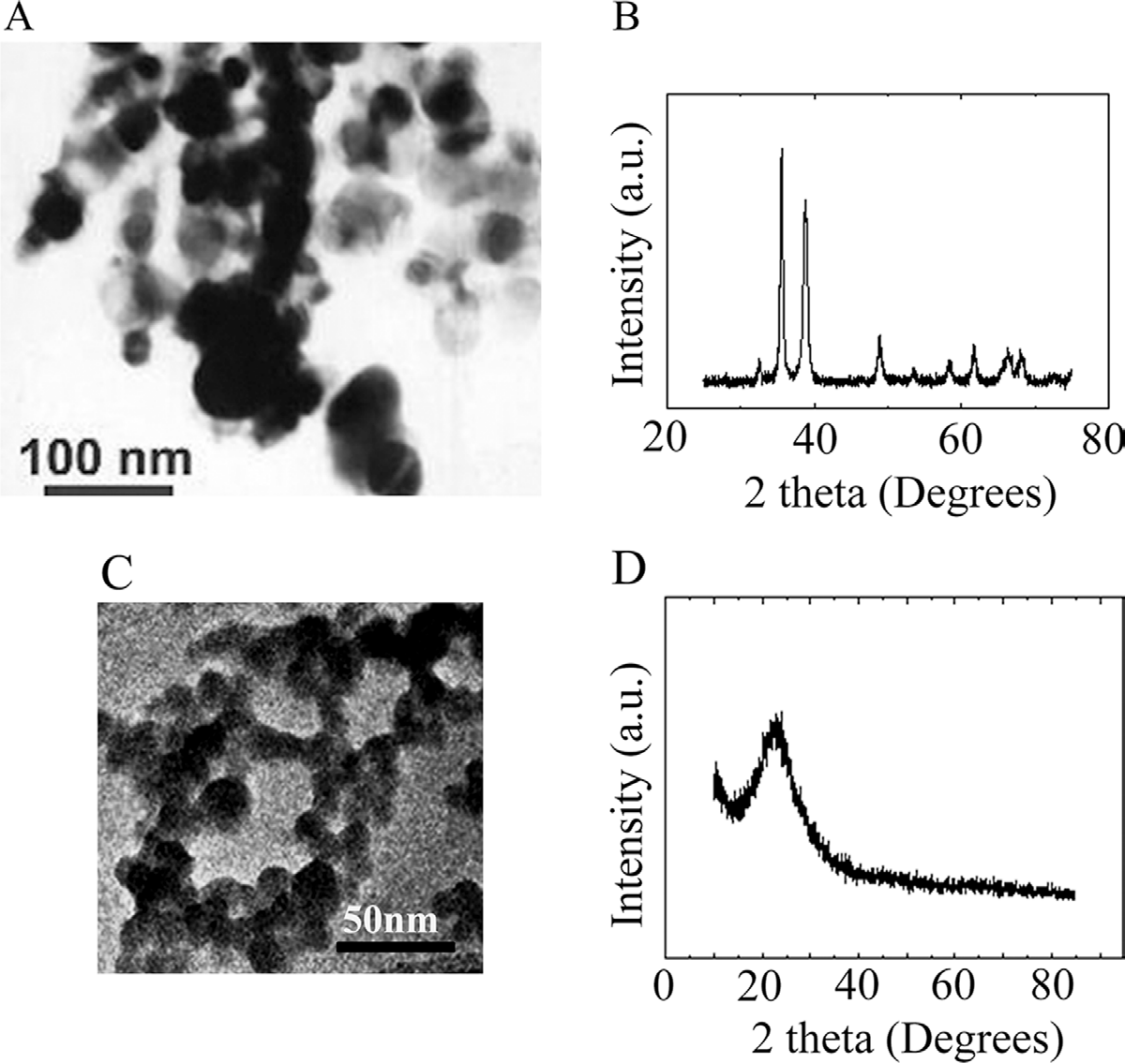
Characterization of CuO and SiO_2_ NPs (**A**) TEM image of CuO NPs. (**B**) XRD image of CuO NPs. (**C**) TEM image of SiO_2_ NPs. (**D**) XRD image of SiO_2_ NPs.

ZnO, CuO, and SiO_2_ NPs were manufactured by Beijing DK Nano Technology Co. LTD (Beijing, China). The ultra-structure of ZnO NPs and their characterization in cells and tissues have been published in our recent articles [Supplementary Figure 1] [80, 81]. Morphologically, the ZnO NPs were nearly spherical with a milky white color. The size, surface area, and density were approximately 30 nm, 50 m^2^/g, and 5.606 g/cm^3^, respectively (Supplementary Figure 1). The hydrodynamic diameter, polydispersity index and zeta potential for ZnO NPs in phosphate buffered saline (PBS) was 148 ± 26 nm, 0.089 μ2/T^2^, or **−**27.6 ± 2.3 mV respectively. The morphology and size of CuO and SiO_2_ NPs were determined using transmission electron microscopy (TEM) and X-ray Diffractometer (Figure 1). The CuO NPs were nearly spherical and black in color. The size, surface area, and density were approximately 30 nm, 13.1 m^2^/g, and 6.4 g/cm^3^, respectively (Figure 1A and 1B). The SiO_2_ NPs were nearly spherical and white in color. Their size, surface area, and density were approximately 30 nm, 600 m^2^/g, and 2.4 g/cm^3^, respectively (Figure 1C and 1D). The hydrodynamic diameter, polydispersity index and zeta potential for CuO NPs in phosphate buffered saline (PBS) was 189 ± 42 nm, 0.072 μ2/T^2^, or **−**43.2 ± 1.7 mV respectively. The hydrodynamic diameter, polydispersity index and zeta potential for SiO_2_ NPs in phosphate buffered saline (PBS) was 137 ± 31 nm, 0.104 μ2/T^2^, or **−**25.4 ± 2.1 mV respectively.

### Inhibition of chicken embryonic development by ZnO NPs

Previously, we found that 10–200 mg/kg (diet) ZnO NPs or 10–200 mg/kg (diet) ZnSO_4_ treatments did not decrease hen body weight or egg production [80]. Intact NPs were detected in ZnO NP treated hen ovarian tissues (Supplementary Figure 1). ZnO NP or ZnSO_4_ treatments had little effect on Zn content in ovarian tissues compared to control treatments (Supplementary Figure 2). The major difference between ZnO NPs and ZnSO_4_ treatments was that the ZnO-NP-200 mg/kg treatment significantly decreased egg yolk lipid content when compared with the ZnSO_4**-**_200 mg/kg treatment [80]. Therefore, ZnSO_4_-200 mg/kg and ZnO-NP-200 mg/kg treatments were used in the current investigation. The concentrations of ZnO NPs or ZnSO_4_ used in our studies were based on the diet. If the concentration of 200 mg/kg of diet was calculated based on animal body weight (BW), it was calculated to be around 20 mg/kg BW. Therefore, the concentration was lower than that used in other embryo-toxic studies (200–400 mg/kg BW) [18, 19].

**Figure 2:**
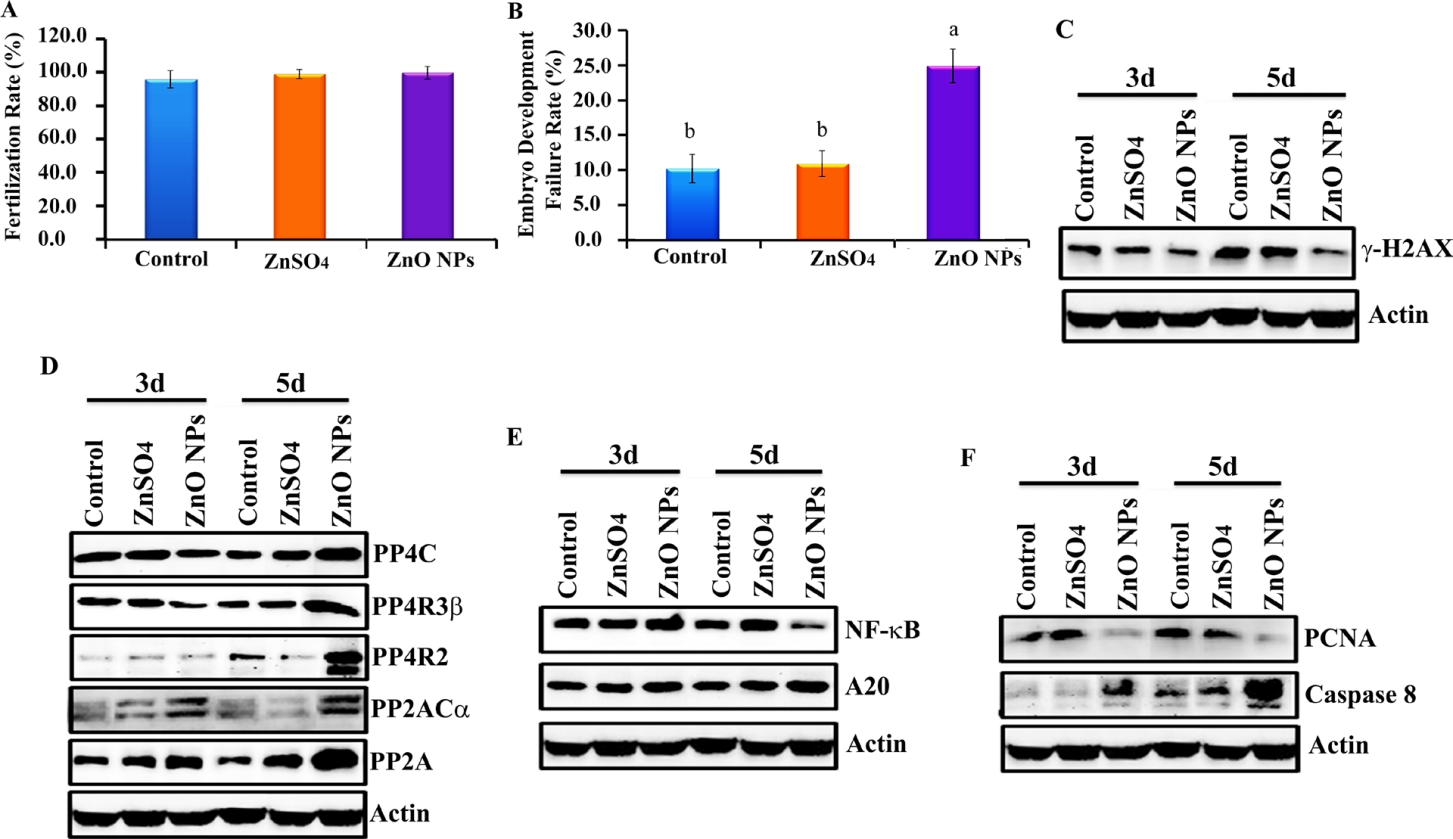
Inhibition of chicken embryonic development by ZnO NPs (**A**) Fertilization rate; (**B**) Embryo development failure rate; (**C**) Decrease in γ-H2AX by ZnO NPs using WB analysis; (**D**) Elevation of the protein levels of de-phosphorylation enzymes PP4C, PP4R3β, PP4R2, PP2ACα, and PP2A by ZnO NPs using WB analysis; (**E**) Reduction in NF-κB major component p65 and increase in A20 by ZnO NPs treatment using WB analysis; (**F**) Decrease in PCNA while elevation in caspase 8 by ZnO NPs treatment using WB analysis; N^3^5.

After a 24-wk trial, hens were artificial inseminated. Before hatching, 200 eggs per treatment were used to determination the fertilization rate; this was approximately 98% and it was the same for control, ZnSO_4_-200 mg/kg and ZnO-NP-200 mg/kg treatments (Figure 2A). However, 7 d after hatching, ZnO-NP-200 mg/kg increased embryonic developmental failure rate (lethality) by 15% when compared with the ZnSO_4_-200 mg/kg treatment or control, which suggested that the ZnO-NP-200 mg/kg treatment inhibited chicken embryonic development (Figure 2B).

### Blockage of γ-H2AX and NF-κB pathways by ZnO NPs in chicken embryos

In order to explore the underlying mechanism of ZnO NP inhibition of chicken embryonic development, the protein levels of many pathways were investigated in 3 d and 5 d embryos. It was found that the protein level of γ-H2AX in embryos was significantly decreased by the ZnO-NP-200 mg/kg treatment (Figure 2C). Phosphorylation enzymes were responsible for the de-phosphorylation of γ-H2AX; therefore, these phosphorylation enzymes were subsequently monitored. It was found that the protein levels of phosphorylation enzymes PP4C, PP4R3β, PP4R2, PP2ACα, and PP2A in embryos were increased by ZnO NPs treatment (Figure 2D). Another important factor, NF-κB, was also reduced by ZnO NPs (Figure 2E). ZnO NPs decreased both NF-κB and γ-H2AX protein levels, which suggested that this treatment might decrease cell proliferation or increase apoptosis. Protein levels of PCNA and apoptosis markers were analyzed by WB. ZnO NPs treatment dramatically reduced PCNA protein levels and increased caspase-8 in chicken embryos (Figure 2F).

### Disturbance in the γ-H2AX pathway due to ZnO NPs in ovarian cells (CKO-K1 cells)

**Figure 3:**
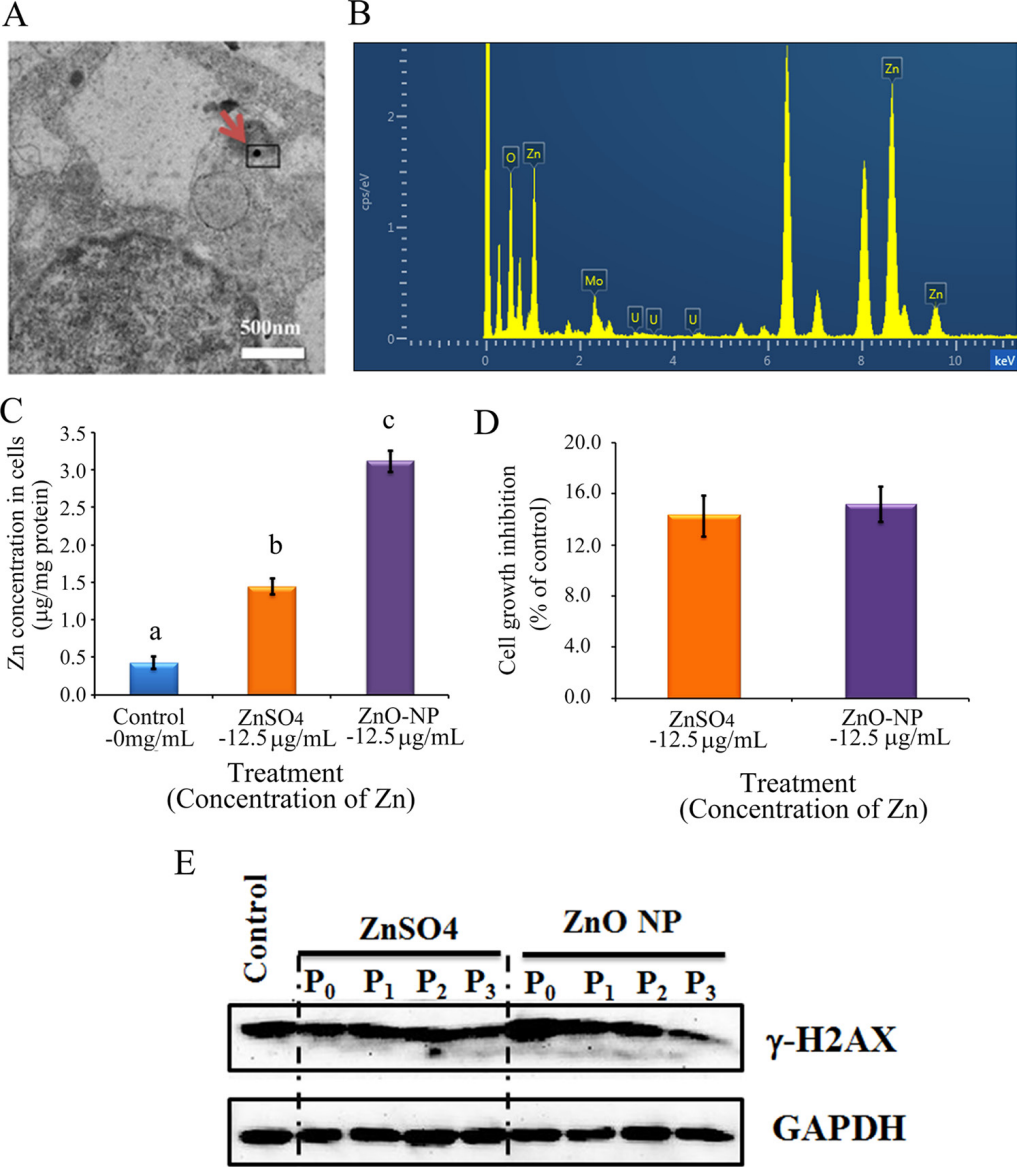
ZnO NPs in CHO-K1 cells and the impacts on cell growth and γ-H2AX (**A**) TEM photo of ZnO NPs in CHO-K1 cells indicated by the red arrow; (**B**) EDS image of ZnO NPs in CHO-K1 cells, three Zn peaks shown; (**C**) Concentration of Zn in ZnO NPs or ZnSO_4_ treated CHO-K1, a,b,c, same letter means no difference, different letters mean the groups have difference, *p* < 0.05; (**D**) Effects of ZnO NP and ZnSO_4_ on CHO-K1 cell growth; (**E**) Alteration of γ-H2AX by ZnO NPs using WB analysis; *N* ≥ 3.

**Figure 4:**
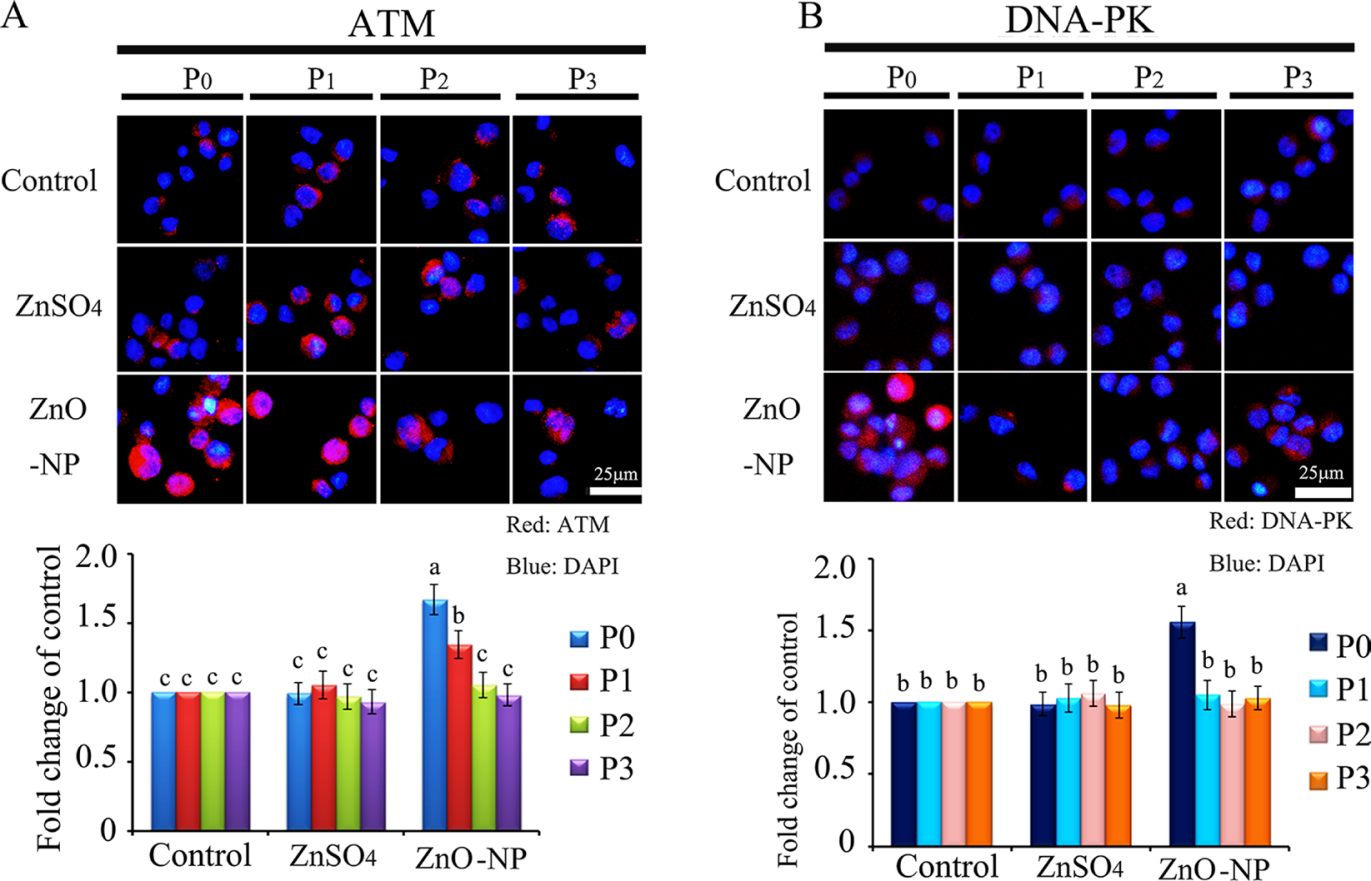
IHF images for ATM and DNA-PK in ZnO NPs treated CHO-K1 cells (**A**) Increase in the protein level of ATM in P_0_ and P_1_ cells by ZnO NPs treatment. Red: ATM staining; Blue: DAPI staining for nucleus; (**B**) Elevation of the protein levels of DNA-PK in P_0_ and P_1_ cells by ZnO NPs treatment. Red: DNA-PK staining; Blue: DAPI staining for nucleus. Scale bar: 25 mm; *N* ≥ 3. ^a-c^ Means not sharing a common superscript are different.

**Figure 5:**
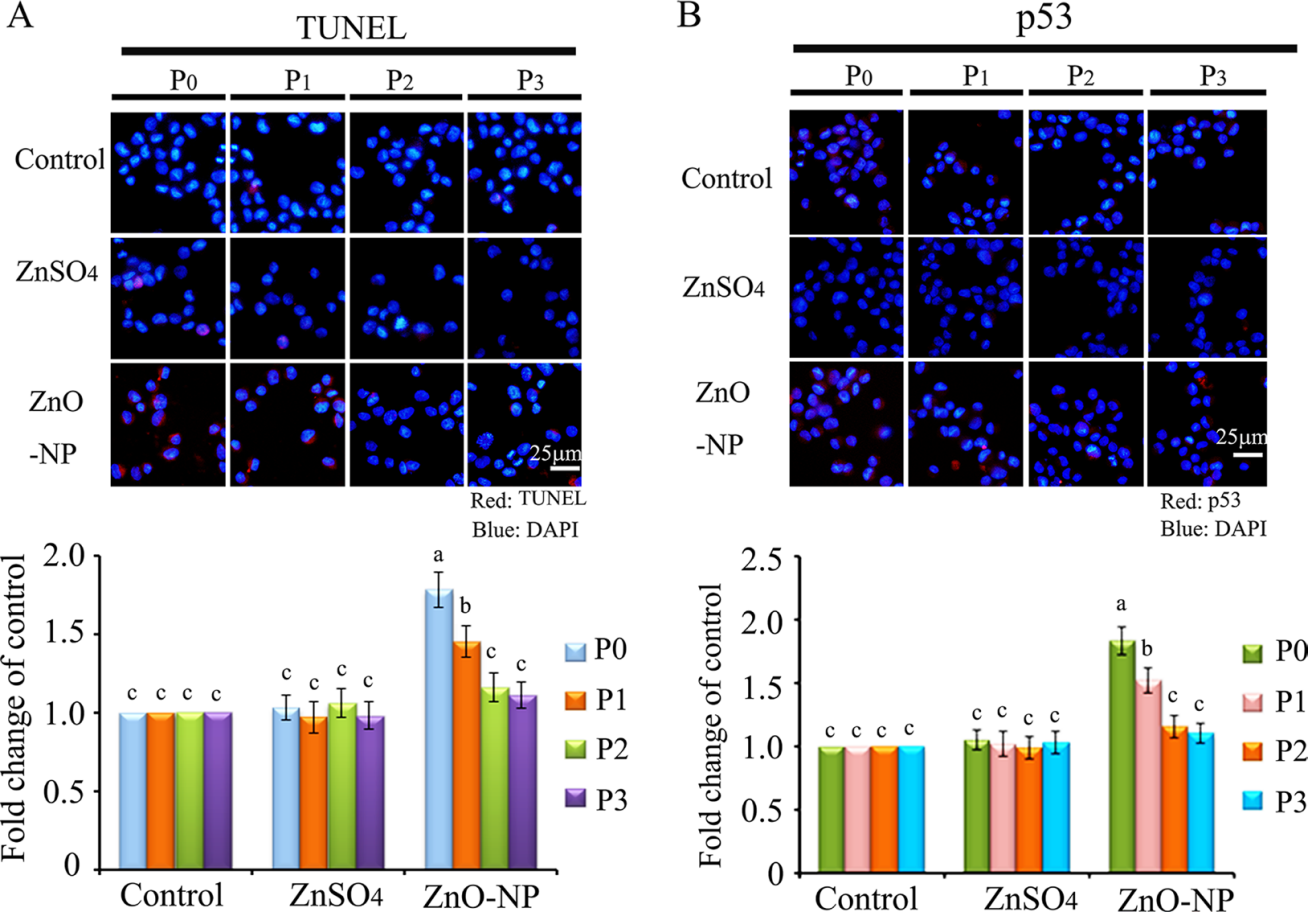
IHF images for TUNEL assay and p53 levels in ZnO NPs treated CHO-K1 cells (**A**) Increase in the TUNEL positive cells in P_0_ and P_1_ cells by ZnO NPs treatment. Red: TUNEL staining; Blue: DAPI staining for nucleus; (**B**) Elevation of the protein levels of p53 in P_0_ and P_1_ cells by ZnO NPs treatment. Red: p53 staining; Blue: DAPI staining for nucleus. Scale bar: 25 μm; *N* ≥ 3. ^a-c^ Means not sharing a common superscript are different.

**Figure 6:**
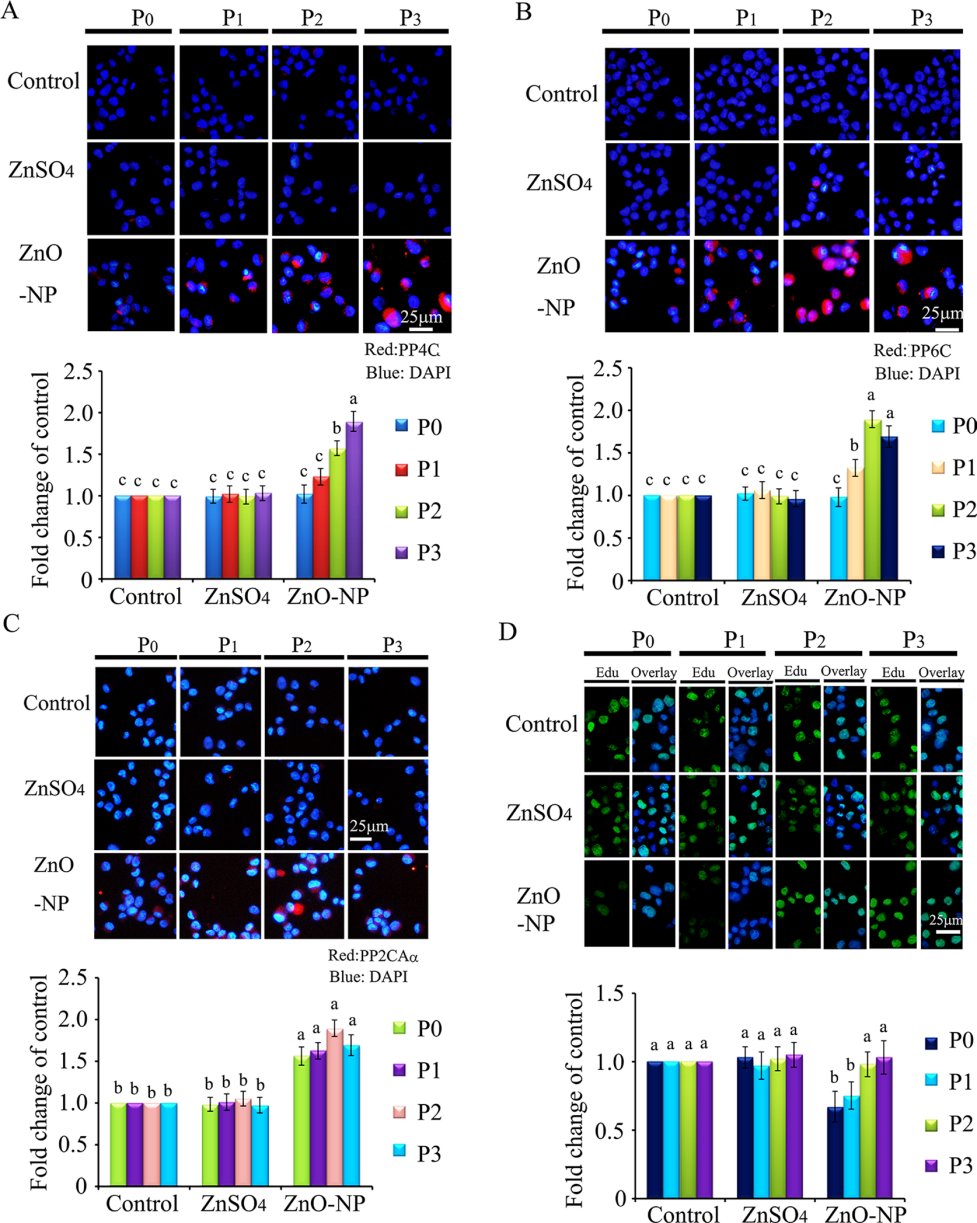
IHF images for de-phosphorylation enzymes PP4C, PP6C, PP2ACα and EdU analysis after ZnO NPs treatment (**A**) IHF staining for PP4C, increase in the PP4C protein levels in P_1_, P_2_ and P_3_ cells. Red: PP4C staining; Blue: DAPI staining for nucleus; (**B**) IHF staining for PP6C, elevation of the PP6C protein levels in P_1_, P_2_ and P_3_ cells. Red: PP6C staining; Blue: DAPI staining for nucleus; (**C**) IHF staining for PP2ACα, increase in the PP2ACα protein levels in P_0_, P_1_, P_2_ and P_3_ cells. Red: PP2ACα staining; Blue: DAPI staining for nucleus; (**D**) EdU analysis, decrease in the EdU positive cells in P_0_ cells. Green: P EdU staining; Blue: DAPI staining for nucleus; Scale bar: 25 mm; *N* ≥ 3. ^a-c^ Means not sharing a common superscript are different.

To verify the phenomenon seen in embryos, hamster ovarian cells (CHO-K1) were cultured and treated with ZnSO_4_-12.5 μg/mL or ZnO-NP-12.5 μg/mL for 24 h. After the 24 h treatment, cells [passage 0 (P_0_)] were collected and half were used for analysis and half were passaged. Following passage and 24 h of growth, the cells [passage 1 (P_1_)] were collected and half were used for analysis and half were passaged again. Subsequently, using the same method, P_2_ and P_3_ cells were collected. Intact NPs were found in ZnO NPs treated CHO-K1 cells (P_0_) and confirmed by EDS (Figure 3A and 3B). The cellular concentration of Zn was increased by ZnSO_4_ and ZnO NPs treatments (P_0_ cells). Further, the concentration of Zn was higher in the ZnO-NP-12.5 μg/mL treatment than that in the ZnSO_4_-12.5 μg/mL treatment (Figure 3C). However, these two treatments produced a similar cell growth inhibition of approximately 15% (Figure 3D). ZnSO_4_ did not stimulate γ-H2AX protein level; however, ZnO NPs significantly increased the protein level of γ-H2AX after 24 h of treatment (P_0_ cells). However, after the passages, γ-H2AX was dramatically reduced (Figure 3E). This indicated that ZnO NPs might cause DNA damage after 24 h treatment. Therefore, the DNA damage related proteins ATM (Ataxia telangiectasia mutated), ATR (ATM- and Rad3-related), and DNA-PK (DNA-dependent protein kinase) were monitored. It was found that ATM and DNA-PK were elevated after a 24-h ZnO NP treatment (P_0_ cells; Figure 4A and 4B); however, they were not changed in P_2_ or P_3_ cells. ATR was not altered by these treatments. Subsequently, TUNEL assay and apoptotic markers were used to monitor apoptosis. More positive cells were detected in ZnO NPs treated cells than that in the control or ZnSO_4_ treatment (Figure 5A) by TUNEL assay. Furthermore, the p53 protein level was also increased by ZnO NPs after 24 h treatment (P_0_; Figure 5B). In P_0_ cells, γ-H2AX was increased to repair the DNA damage. However, γ-H2AX was decreased gradually from P_1_ to P_3_ cells to lower than that in the control treatment, which indicated that γ-H2AX itself might also be suppress by ZnO NPs. Phosphorylation enzymes were also determined and it was found that PP4C and PP6C were gradually elevated from P_1_ to P_3_ cells and PP2ACα was also increased from P_0_ to P_3_ cells in ZnO NPs treatment (Figure 6A–6C). The proliferation of the cells was determined by EdU assay and it was found that EdU positive cells were decreased in P_0_ and P_1_ cells (Figure 6D) due to ZnO NPs treatment. However, ZnSO_4_ did not induce DNA damage, apoptosis rate, or γ-H2AX protein level.

### Inhibition of the NF-κB pathway by ZnO NPs in CKO-K1 cells

**Figure 7:**
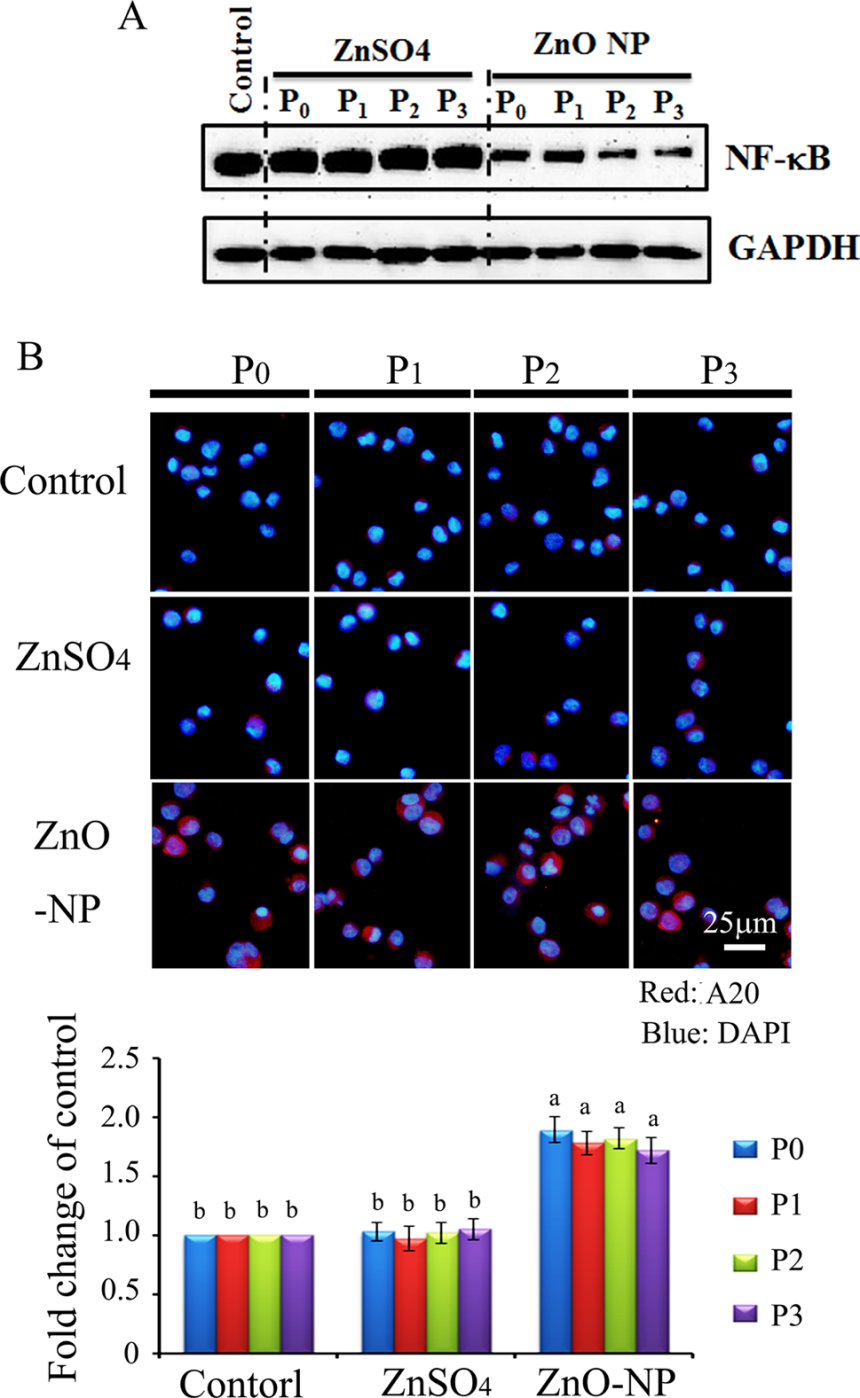
NF-κB major component p65 and A20 in ZnO NPs treated CHO-K1 cells (**A**) reduction in NF-κB major component p65 by ZnO NPs treatment; (**B**) Increase in A20 by ZnO NPs in CHO-K1 cells using IHF staining; Scale bar: 25 mm; *N* ≥ 3. ^a-b^ Means not sharing a common superscript are different.

Protein levels of NF-κB (p65) were also detected using WB. It was found that NF-κB (p65) was significantly and similarly decreased by the ZnO NPs treatment of P_0_ to P_3_ cells. However, ZnSO_4_ did not alter NF-κB in all these passaged cells (Figure 7A). A20 (TNFAIP3) was found to be an inhibitor of NF-κB, and it was found that ZnO NPs dramatically increased A20 in all passaged cells (P_0_ to P_3_; Figure 7B). However, A20 was not altered in ZnSO_4_ treated cells. The data here might suggest that ZnO NPs treatment blocked the NF-κB pathway through A20.

### Impairment of γ-H2AX not NF-κB by CuO or SiO2 NPs in CHO-K1 cells

**Figure 8:**
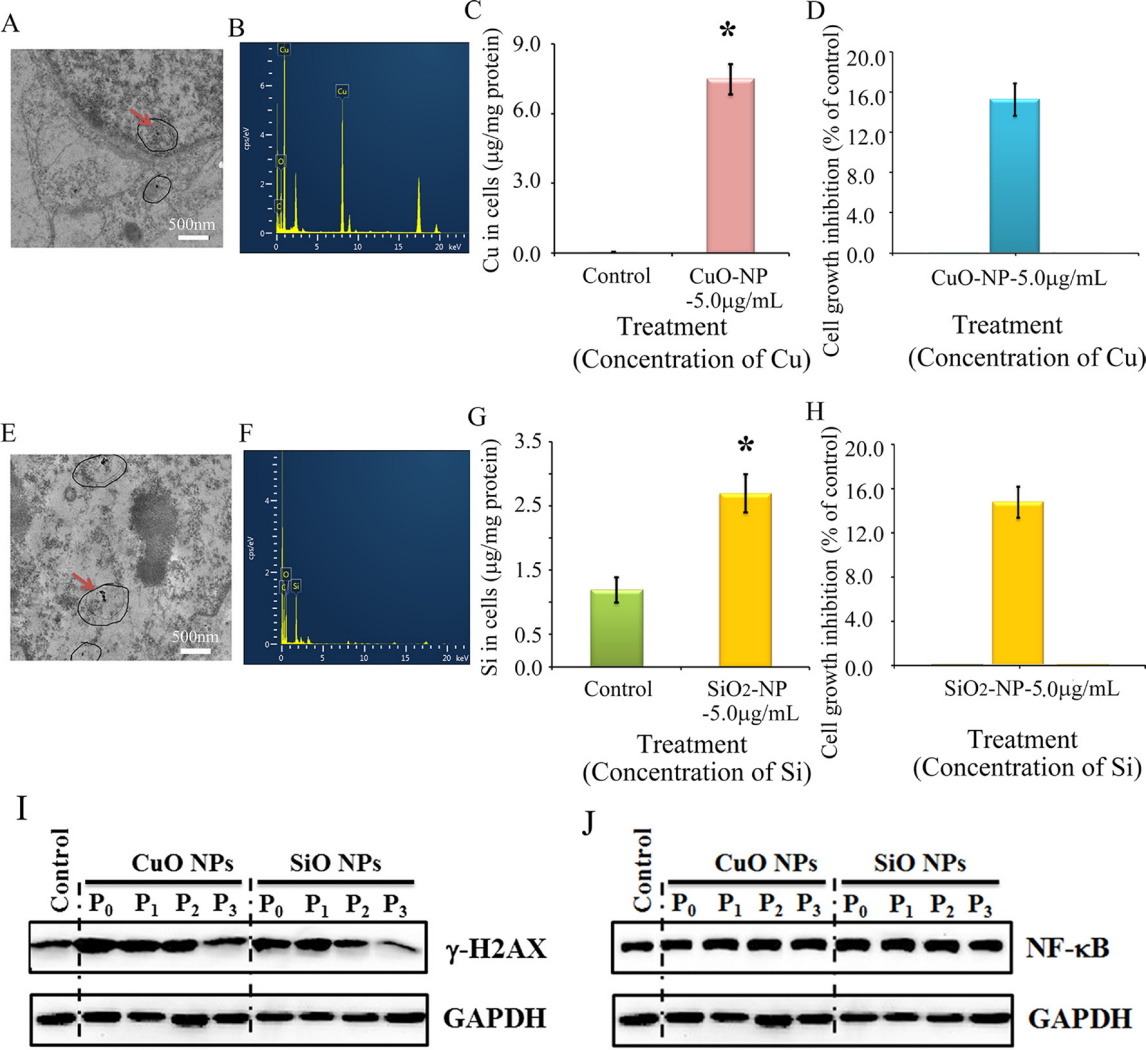
Impairment of γ-H2AX not NF-κB by CuO or SiO2 NPs in CHO-K1 cells (**A**) TEM photo of CuO NPs in CHO-K1 cells indicated by the red arrow; (**B**) EDS image of CuO NPs in CHO-K1 cells, two Cu peaks have shown; (**C**) Concentration of Cu in CuO NPs treated CHO-K1, *p < 0.05; (**D**) Effects of CuO NPs on CHO-K1 cell growth; (**E**) TEM photo of SiO_2_ NPs in CHO-K1 cells indicated by the red arrow; (**F**) EDS image of SiO_2_ NPs in CHO-K1 cells; (**G**) Concentration of Si in SiO_2_ NPs treated CHO-K1, *p < 0.05; (**H**) Effects of SiO_2_ NPs on CHO-K1 cell growth; (**I**) Decrease in γ-H2AX by CuO and SiO_2_ NPs using WB analysis; (**J**) No alteration in NF-κB major component p65 by CuO NPs or SiO_2_ NPs treatment using WB analysis; *N* ≥ 3.

It is still not clear, and it is even controversial whether the toxic effects of ZnO NPs come from Zn^2+^ or intact nanoparticles. The properties of CuO NPs and ZnO NPs are similar as they can be dissolved in acidic or neutral solutions to release ionic forms of Zn or Cu. However, SiO_2_ NPs differ in that they cannot be dissolved in acidic or neutral solutions. Therefore CuO NPs and SiO_2_ NPs were used to verify the impact of ZnO NPs. If CuO and SiO_2_ NPs produce similar effects to ZnO NPs, this would suggest that the effects of the latter come from intact nanoparticles; if similar results are not achieved, it would suggest that the effects of ZnO NPs come from Zn^2+^. Intact CuO NPs were detected in treated cells (Figure 8A) and confirmed by EDS (Figure 8B). After a 24-h treatment, the copper content in the CuO-NP-5 μg/mL treatment was much higher than that in the control cells (Figure 8C). Figure 8E showed intact SiO_2_ NPs in CHO-K1 cells after a 24-h treatment and this was confirmed by EDS (Figure 8F). The content of Si in the SiO_2_-NP-5 μg/mL treatment was higher than that in the control cells (Figure 8G). Cell growth inhibition was similar (16%) for both CuO-NP-5 μg/mL and SiO_2_-NP-5 μg/mL treatments (Figure 8D and 8H). γ-H2AX was elevated by CuO-NP-5 μg/mL and SiO_2_-NP-5μg/mL after 24 h treatments (P_0_ cells); subsequently it was gradually decreased during passages (from P_1_ to P_3_ cells; Figure 8I). However, NF-κB was not altered by the CuO-NP-5 μg/mL or SiO_2_-NP-5 μg/mL treatment, and a similar level was maintained during passages (from P_0_ to P_3_ cells; Figure 8J).

**Figure 9:**
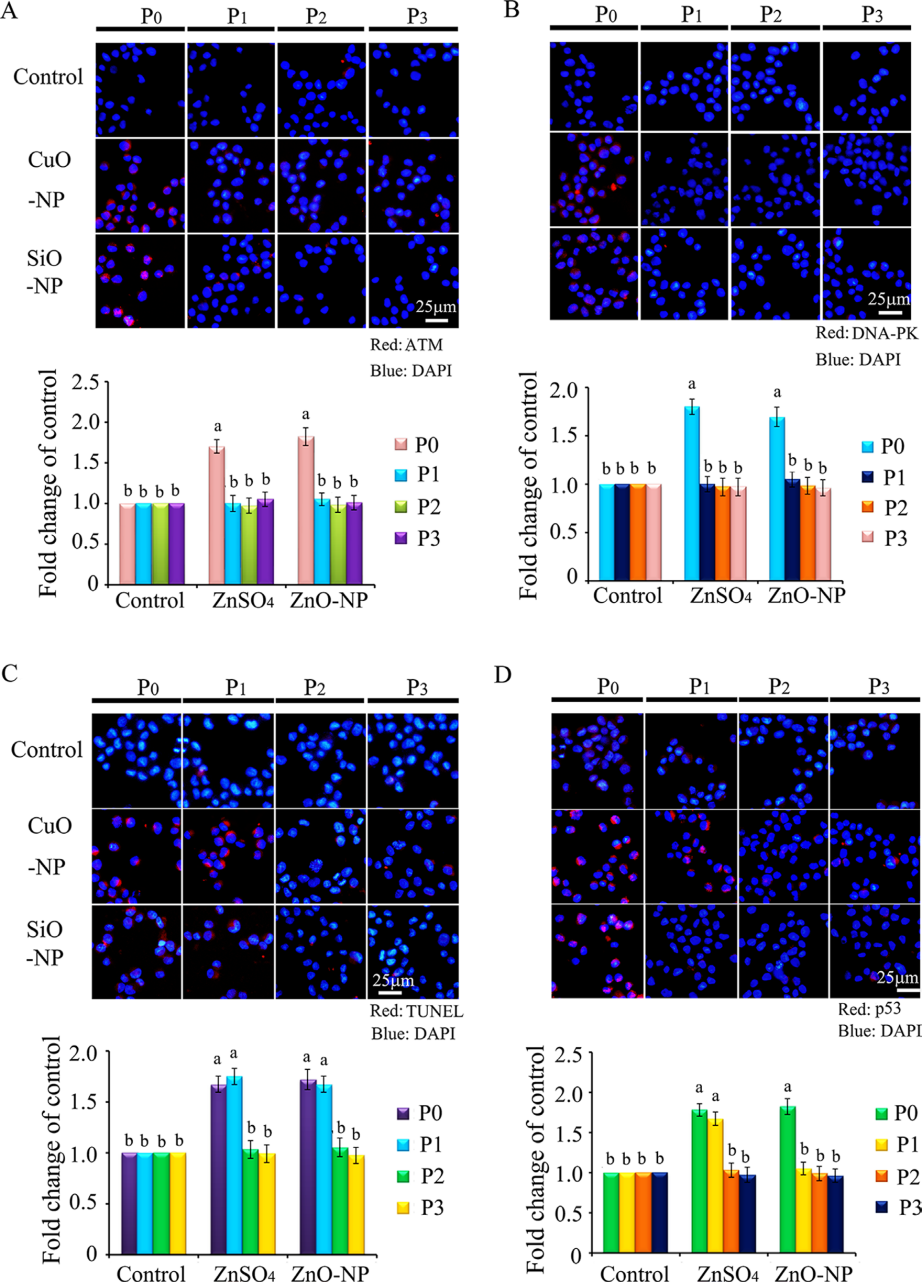
IHF images for ATM, DNA-PK, TUNEL assay and p53 levels in CuO and SiO2 NPs treated CHO-K1 cells (**A**) Increase in the protein levels of ATM in P_0_ and P_1_ cells. Red: ATM staining; Blue: DAPI staining for nucleus; (**B**) Elevation of the protein levels of DNA-PK in P_0_ and P_1_ cells. Red: DNA-PK staining; Blue: DAPI staining for nucleus. Scale bar: 25 μm. (**C**) Increase in TUNEL positive cells in P_0_ and P_1_ cells. Red: TUNEL staining; Blue: DAPI staining for nucleus; (**D**) Elevation of the protein levels of p53 in P_0_ and P_1_ cells. Red: p53 staining; Blue: DAPI staining for nucleus. Scale bar: 25 μm; *N* ≥ 3. ^a-b^ Means not sharing a common superscript are different.

Since γ-H2AX was altered by CuO-NP-5 μg/mL and SiO_2_-NP-5 μg/mL treatments, ATM, ATR and DNA-PK were determined in CHO-K1 cells after treatment. It was found that ATM and DNA-PK were increased in P_0_ cells in both CuO-NP-5 μg/mL and SiO_2_-NP-5 μg/mL treatments (Figure 9A and 9B). TUNEL positive cells were found at higher levels in P_0_ and P_1_ cells, in both the CuO-NP-5 μg/mL and SiO_2_-NP-5 μg/mL treatments, than that in the control treatment (Figure 9C). The protein level of p53 was higher in P_0_ and P_1_ cells in the CuO-NP-5 μg/mL treatment than that in the control treatment, while it was higher in P_0_ cells in the SiO_2_-NP-5μg/mL treatment than that in the control (Figure 9D).

**Figure 10:**
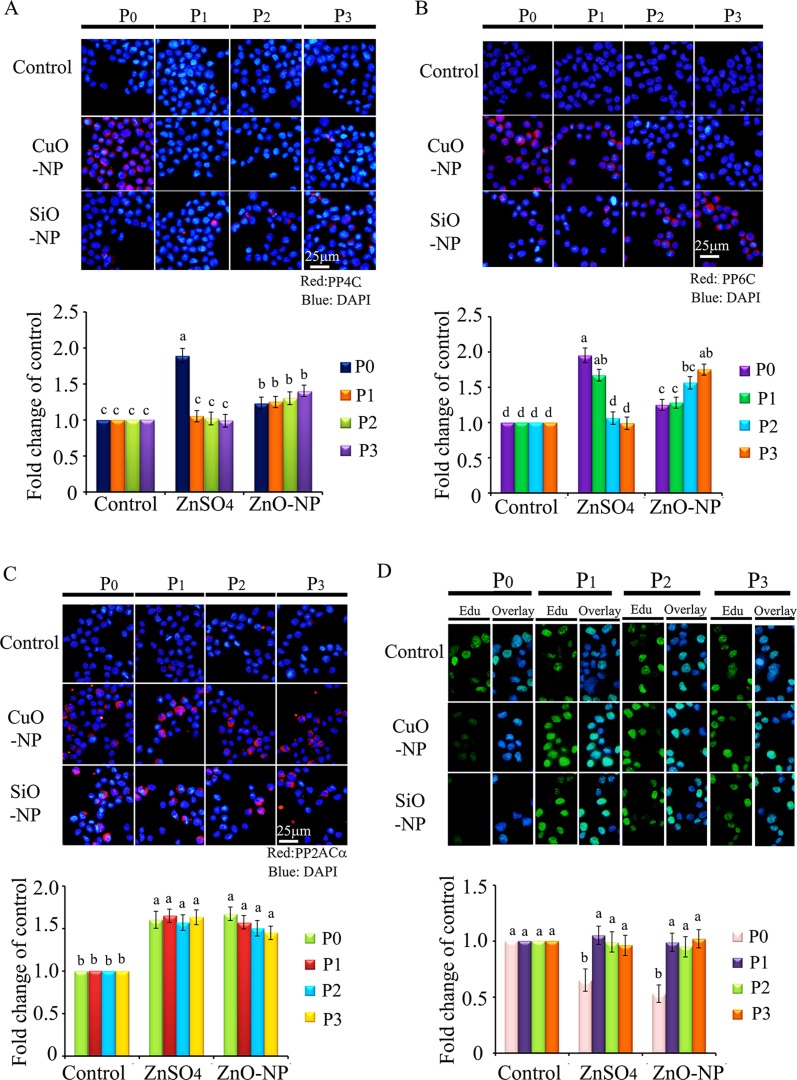
IHF images for de-phosphorylation enzymes PP4C, PP6C, PP2ACα and EdU analysis in CuO and SiO2 NPs treated cells (**A**) IHF staining for PP4C, increase in the PP4C protein levels in P_1_, P_2_ and P_3_ cells. Red: PP4C staining; Blue: DAPI staining for nucleus; (**B**) IHF staining for PP6C, elevation of the PP6C protein levels in P_1_, P_2_ and P_3_ cells. Red: PP6C staining; Blue: DAPI staining for nucleus; (**C**) IHF staining for PP2ACα, increase in the PP2ACα protein level in P_0_, P_1_, P_2_ and P_3_ cells. Red: PP2ACα staining; Blue: DAPI staining for nucleus; (**D**) EdU analysis, decrease in the EdU positive cells in P_0_ cells. Scale bar: 25 μm; *N* ≥ 3. ^a-d^ Means not sharing a common superscript are different.

Protein levels of PP4C, PP6C, and PP2ACα were elevated in both CuO-NP-5 μg/mL and SiO_2_-NP-5 μg/mL treatments (Figure 10A–10C). EdU positive cells were decreased in P_0_ cells in both CuO-NP-5 μg/mL and SiO_2_-NP-5 μg/mL treatments (Figure 10D). Altogether, ZnO-NP-12.5 μg/mL, CuO-NP-5 μg/mL, and SiO_2_-NP-5 μg/mL treatments altered γ-H2AX in a similar pattern in the four passages cells (P_0_ to P_3_); however, only the ZnO-NP-12.5 μg/mL treatment decreased NF-κB in the four passages cells (P_0_ to P_3_). These findings suggest that intact NPs from ZnO, CuO, and SiO_2_ NP treatments block the γ-H2AX pathway while just Zn^2+^ upsets the NF-κB pathway. Clearly, the toxic effects of ZnO NPs emanate from both intact nanoparticles and Zn^2+^. These *in vitro* culture findings matched well with *in vivo* embryonic data, suggesting that impairment caused by ZnO NP treatment can pass through cell generations, and that the γ-H2AX and NF-κB pathways were involved in the embryo-toxicity of ZnO NPs.

## DISCUSSION

Researchers have explored the effects of nanoparticles on embryo development; however, these studies have only investigated the effects (phenomenon) on embryos [1–71], and not on oocytes. Therefore, the impacts of nanoparticles on embryonic development due to oocyte stage exposure are as yet unknown, and the underlying mechanisms are sparsely understood. ZnO NPs are commonly used in almost every aspect of our lives especially in sunscreen, cosmetics, and biocides [72–76]. Even though many reports indicate that ZnO NPs cause adverse effects on reproductive systems and embryonic development [3, 7, 14, 15, 18–20, 26, 40, 47, 62], their safety is not fully understood, particularly the impacts and underlying mechanisms of ZnO NPs on embryonic development due to oocyte stage exposure.

In the current investigation, hens were exposed to ZnO NPs, and after fertilization their impacts on embryonic development and the underlying mechanisms were explored. Results indicated that ZnO NPs inhibited embryonic development by increasing embryo lethality; however, the fertilization rate was not suppressed. Further mechanistic study found that γ-H2AX in embryos was decreased with ZnO NPs treatment. It has been reported that nanoparticles can induce ROS and DNA damage, which consequently activate ATM to induce H2AX phosphorylation (γ-H2AX) [82–85]. ZnO NPs induce DNA damage and increase γ-H2AX to inhibit cell growth [82, 85]. However, many recent investigations suggest that γ-H2AX is not simply a specific DNA DSB marker and its role is not restricted to the DNA damage response [86]. Reports suggest that it involves a multitude of biological processes during cell division [86, 87], stem cell biology [88–90], angiogenesis [91–93], and aging [94–96]. The de-phosphorylation of γ-H2AX by phosphatases is an important event in complete DNA repair after exogenous DNA damage [97–100]. Two families of phosphatases are involved in the de-phosphorylation of γ-H2AX: firstly, the PP2A family of serine/threonine phosphatases including four distinct catalytic proteins PP2ACα, PP2ACβ, PP4C, and PP6C [97, 99], and secondly the PP4 phosphatase complex containing PP4C, PP4R2, and PP4R3β [97, 99]. In the current study, these phosphatases were determined, and it was found that PP4C, PP4R3β, PP4R2, PP2ACα, and PP2A were elevated by ZnO NPs in embryos (Figure 2D). Transcription factor NF-κB (nuclear factor-kappa B) is a critical regulator of multiple biological functions: cell growth, cell survival, innate and adaptive immunity, and others [101–104]. In the current study it was found that ZnO NPs treatment decreased NF-κB in embryos. Vyas *et al*. (2015) found that iron oxide nanoparticles could enhance the effects of the anticancer drug, doxorubicin, through elevation in apoptosis and down regulation of both pro-inflammatory mediators IL-6 and NF-κB [105]. Sarkar *et al*. (2015) reported that Ag NPs exposure suppressed mycobacterium tuberculosis-induced expression of a subset of NF-κB mediated genes to suppress mycobacterium tuberculosis-induced NF-κB activation and host immune responses in macrophage cells [106]. A20 (TNFAIP3) has been originally described as a zinc finger protein which is a TNF-inducible gene and acts to protect cells from TNF cytotoxicity [107, 108]. During later experiments, it has been found that A20 acts as a negative regulator of NF-κB signaling involving IL-1b, TNFα, TRAF1/TRAF2, RIP/TRAF2, and other factors [109–111]. Kim and Jeong (2015) reported that ZnO NPs induced the expression of A20 and reduce NF-κB activation to increase anti-inflammatory effects [112]. Feng *et al*. (2016) found that A20 suppressed the expression of inflammatory cytokines induced by Ag NPs [113]. In the current study, ZnO NP treatment elevated A20 and decreased NF-κB in embryos. Since the NF-κB pathway is very important for cell survival and growth, the blockage of this pathway might be one of the major reasons for the inhibition of embryonic development caused by ZnO NPs.

ZnO NPs exposure may damage DNA replication and repairmen machinery in hen oocytes, which impairs the embryos to inhibit embryonic development. In order to confirm this phenomenon, normal hamster ovarian cells (CHO-K1) were used to determine the impact of ZnO NPs exposure on γ-H2AX and NF-κB pathways after a 24-h treatment and three cell passages. γ-H2AX was elevated after a 24-h ZnO NP exposure, but it was gradually decreased during progressive cell passages to a level much lower than that in the control treatment; this indicated that ZnO NP treatment caused DNA damage and DNA repairmen/replication machinery failure. Furthermore, the phosphatases PP4C, PP6C, and PP2ACα were induced in P_2_ and P_3_ cells, which coordinated well with changes of γ-H2AX. NF-κB was diminished after a 24-h ZnO NPs exposure and remained at similar low levels during cell passages while A20 was present at higher levels in all passaged cells than in the control treatment. These *in vitro* results verified that ZnO NPs treatment blocked both γ-H2AX and NF-κB pathways *in vivo* (embryos).

ZnO NPs can be endocytosed into cells and dissolved to release Zn^2+^ to significantly increase intracellular Zn^2+^ levels [114, 115]. It is known that locally high levels of intracellular Zn^2+^ have toxic effects [114, 115]. Furthermore, ZnO NPs internalized into cells remain as intact NPs for a long time and these induce different toxic effects when compared with Zn^2+^ [116–119]. We have already found that ZnO NPs and ZnSO_4_ (sole Zn^2+^ provider) produced a significantly different impact on gene and protein expression *in vitro* and *in vivo* with intact NPs being detected in cells and animal tissues [117–119]. Intact ZnO NPs can be found in hen ovarian, liver, spleen, and uterine tissues [80, 81, 117–119]. In order to investigate whether the blockage of γ-H2AX and NF-κB pathways by ZnO NP treatment was due to intact NPs or Zn^2+^, CuO NPs and SiO_2_ NPs were used in the current investigation. The size of ZnO, CuO, and SiO2 NPs is similar at around 30 nm. The properties of CuO NPs are similar to ZnO NPs in that they can be dissolved in cells, animal tissues, or even solutions [25, 64]. However, SiO_2_ NPs are different from ZnO and CuO NPs in that SiO_2_ NPs cannot be dissolved *in vivo* or *in vitro* culture [63]. If ZnO, CuO, and SiO_2_ NPs produce similar effects on γ-H2AX and NF-κB pathways, it would suggest that these effects are from intact NPs; if not, the effects would emanate from Zn^2+^. In the current study it was found that γ-H2AX was increased in CHO-K1 cells after 24 h CuO NP and SiO NP treatments, and it was decreased with progressive cell passages; a similar trend was seen with ZnO NP treatment. This indicated that γ-H2AX blockage was most likely caused by intact NPs (ZnO, CuO, or SiO_2_ NPs). However, NF-κB was not altered by CuO NPs or SiO_2_ NPs treatments, which suggested that NF-κB blockage was due to Zn^2+^. It seems clear that the toxic effects of ZnO NPs emanate from both intact NPs and Zn^2+^. The blockage of γ-H2AX and NF-κB pathways might increase apoptosis and reduce cell proliferation as indicated by the apoptosis markers and cell proliferating assay in ZnO NP, CuO NP, and SiO NP treatments.

## CONCLUSIONS

In summary, intact NPs were detected in ovarian tissue *in vivo* and cultured cells *in vitro* under ZnO NPs treatments. ZnO NPs treatment inhibited embryo development following oocyte stage exposure, and the γ-H2AX and NF-κB pathways involve in the inhibition of embryo development. Notably, the *in vitro* cultural data closely matched *in vivo* embryonic data indicating that impairments caused by ZnO NPs treatment can pass through cell generations, and that γ-H2AX and NF-κB pathways were involved in the embryotoxicity of ZnO NPs. The current study clearly shows that the toxic effects of ZnO NPs emanate from both intact NPs and Zn^2+^. This study, along with others, suggests that ZnO NPs are toxic to the female reproductive system [ovaries (oocytes)] and subsequently embryo-toxic. Therefore, precautions should be taken against human exposure during daily life.

## MATERIALS AND METHODS

### Characterization of ZnO, CuO and SiO_2_ NPs

ZnO NPs were synthesized by Beijing DK Nano Technology Co. LTD (Beijing, China) as reported in our recent publications [80, 81]. The characteristics of ZnO NPs (morphology, size, agglomeration, etc.) were determined by transmission electron microscopy (TEM; JEM-2100F, JEOL Inc., Japan) and dynamic light scattering (DLS) particle size analyzer (Nano-Zetasizer-HT, Malvern Instruments, Malvern, UK). CuO and SiO2 NPs were manufactured by Beijing DK Nano Technology Co. LTD (Beijing, China) too. The morphology and size of CuO and SiO_2_ NPs were characterized using transmission electron microscopy (TEM; JEM-2100F, JEOL Inc., Japan) and a D8 Advance Powder X-ray Diffractometer (Bruker AXS, Karlsruhe, Germany). The hydrodynamic diameter, polydispersity index and zeta potential were determined in phosphate buffered saline (PBS) after 30 min sonication.

### Animal study design (diets and treatments) and sample collection

All animal experimental procedures followed the regulations of the animal ethics committee of the Qingdao Agricultural University and were reported in our recent publication [80]. All hens (Jinghong-1 strain) were housed in a ventilated and conventional caged commercial poultry house with a lighting program of 16:8 light/dark and ad-lib food and water. The formulation of the basal diet (corn-soybean base) has been previously reported [Supplementary Table 1] [80, 118]. The experimental feeding time was from 6 wks to 32 wks of age (Supplementary Figure 3A). ZnO NPs or ZnSO_4_ was added into the diet and the concentration of Zn (mg/kg) addition was based on diet. The two treatments were ZnSO_4_-200 mg/kg and ZnO-NP-200 mg/kg of diet. A total of 400 pullets were randomly assigned to the two treatments, with five replicates per treatment and forty animals per replicate. After 24 wks treatments, the hens were artificially inseminated with fresh, diluted semen 0.03 mL/hen providing about 210 million sperm (Supplementary Figure 3A). Eggs were collected and stored at 13°C and 75% relative humidity for 5 days until placed in incubators. The fertilization rate was detected by propidium iodide staining of the germinal disc. Egg yolks were separated from the albumen and placed into 0.9% NaCl solution. After visual fertility examination germinal discs seeming to be infertile were removed from the vitelline membrane, put into 0.9% NaCl solution and stained on a slide with propidium iodide (PI; P4170, Sigma-Aldrich Ltd). In case of fertile egg, propidium iodide stains the nucleus of the dividing embryonic cells which appear in lighting points [120, 121]. The embryonic development failure rate was determined by candling eggs after 7 d of incubation and breaking out eggs to differentiate early dead from infertile [120–122]. At 3 d and 5 d incubation, 50 embryos/treatment were collected and stored at **−**80°C. At the age of 30, 31 and 32 wks, the experiments were repeated three times.

### CHO-K1 cell culture

The Hamster Ovarian cell line CHO-K1 was obtained from the American Type Culture Collection (ATCC) (Manassas, VA, USA) and was maintained in Dulbecco's modified Eagle's medium (DMEM) supplemented with 10% FBS, 1 mM sodium pyruvate, 100 U/mL penicillin, and 100 μg/mL streptomycin (Gibco Invitrogen Corporation, USA) at 37°C in 5% CO_2_ [123].

### *In vitro* cytotoxicity assays

CHO-K1 cells were plated in 96-well plates at a density of 5000 cells/well. After 24h cell attachment and growth, the cells were treated with different concentrations (based on Zn, Cu or Si; 0.1, 1.0, 5.0, 7.5, 10.0, 12.5 15.0, 20.0, 30.0 and 50.0 μg/mL) of ZnO NPs, ZnSO_4_ (Cat. No: Z1001, Sigma-Aldrich Co. LLC in China, Beijing, P.R. China), CuO NPs, and SiO_2_ NPs for 24 h. After 24 h treatment, the cells were washed with fresh basic medium (No FBS or antibiotics) and then the cell viability was determined by the reported method using a colorimetric assay with MTT [3-(4, 5)-dimethylthiazol-2, 5-diphenyltetrazolium bromide; Cat. No: M5655, Sigma-Aldrich [81].

### *In vitro* mechanism study

CHO-K1 were cultured and treated with ZnSO_4_, ZnO NPs, CuO NPs or SiO_2_ NPs for 24 h (Supplementary Figure 3B). After 24h treatment, the cells (passage 0 (P_0_)) were collected and half were used for analysis and half were passaged. Following passage and 24 h growth the cells (passage 1 (P_1_)) were collected and half were used for analysis and half were passaged. Similarly, P_2_ and P_3_ cells were collected (Supplementary Figure 3B). The cells collected were for detection of NPs presentation in cells, cellular element concentrations, protein levels by western blotting or IHF analysis.

### Detection of ZnO NPs in cultural cells using transmission electron microscopy (TEM) and energy disperse spectroscopy (EDS)

Sample preparation procedures for detecting nanoparticles are reported in our recent publication [80, 81]. Briefly, cell samples were collected and fixed for 2 h in 2% glutaraldehyde made in sodium phosphate buffer (pH 7.2). Specimens were then washed extensively to remove the excess fixative and subsequently post-fixed in 1% OsO_4_ for 1 h in the dark. Specimens were then dehydrated in an increasingly graded series of ethanol and infiltrated with increased concentrations of Spur's embedding medium in propylene epoxide. Subsequently the specimens were polymerized in embedding medium for 12 h at 37°C, 12 h at 45°C, and 48 h at 60°C. Fifty nanometer sections were cut on a Leica Ultracut E equipped with a diamond knife (Diatome, Hatfield, PA), and collected on form var-coated, carbon-stabilized Mo grids. The section-containing grids were stained with uranyl acetate, air dried overnight, and imaged on a JEM-2010F TEM (JEOL Ltd., Japan). The presence of ZnO NPs in the tissues was confirmed by using X-Max^N^ 80 TLE EDS (Oxford Instruments, U.K.).

### Measurement of Zn concentration in cultured cells

After 24 h treatment with ZnO NPs, ZnSO_4_, CuO NPs or SiO_2_ NPs, cells (2 × 10^6^ cells/treatment) were lysed in 500 μL of 0.4% Triton X-100 in PBS, then the lysate was diluted to 2mL with 0.1% Triton X-100. Samples were determined by inductively coupled plasma optical emission spectroscopy (ICP-OES, Optima 2100, Perkin-Elmer, Shelton, CT, USA) [81]. The voltage for the ion lens was set at 6 V; the gas flow rate in the spray chamber was 0.88 L/min; the power output for the RF generator was 1100 W; the auxiliary gas flow rate was 1.2 L/min; and the nebulizer gas flow rate of the plasma was 16 L/min. All the certified reference materials (in solution) were purchased from the National Institute of Metrology (Beijing, China). Blank controls underwent the same procedures. All procedures were performed in triplicate [81].

### EdU (5-Ethynyl-2′-deoxyuridine) cell proliferation assay

After 22 h ZnSO_4_, ZnO NPs, CuO NPs and SiO_2_ NPs treatment, a Click-iT 5-ethynyl-2′-deoxyuridine (EdU) kit (Molecular Probes, Carlsbad, CA, USA) was used to measure cell proliferation according to the manufacturer's procedures [124]. Cells were labeled with EdU (10 μmol/L) for 2 h. After fixation (4% paraformaldehyde, 60 minutes) and transparency (0.5% Triton X-l00, 30 minutes) treatment, the cells were incubated with Click-iT^®^ reaction cocktail, followed by 4′,6-diamidino-2-phenylindole (DAPI) nuclear staining. After rinsing three times, cells were observed under an inverted fluorescence microscope with three random fields of view. The stained sections were visualized with a Nikon Eclipse TE2000-U fluorescence microscope (Nikon, Inc., Melville, NY), and the captured fluorescent images were analyzed using MetaMorph software [118].

### TUNEL assay

The apoptotic cells were determined by the Dead End Colorimetric TUNEL assay kit (Promega, Madison, WI, USA) with CHO-K1 cells after ZnSO_4_, ZnO NPs, CuO NPs and SiO_2_ NPs treatments according to the manufacturer's protocols. This assay is detecting the DNA fragmentation by labeling the terminal end of nucleic acids. The stained sections were visualized with a Nikon Eclipse TE2000-U fluorescence microscope (Nikon, Inc., Melville, NY), and the captured fluorescent images were analyzed using MetaMorph software [118].

### Western blotting

Embryo and CHO-K1 cell samples were lysed in RIPA buffer containing a protease inhibitor cocktail from Sangong Biotech, Ltd. (Shanghai, China). Protein concentration was determined using a BCA kit (Beyotime Institute of Biotechnology, Shanghai, PR China) [81]. The information for the primary antibodies (Abs) is present in Supplementary Table 2. GAPDH and Actin were used as loading controls. Secondary donkey anti-goat Ab (Cat no. A0181) was purchased from Beyotime Institute of Biotechnology, and goat anti-rabbit (Cat no.: A24531) Abs were bought from Novex^®^ by Life Technologies (USA). Fifty micrograms of total protein per sample were loaded onto 10% SDS polyacrylamide electrophoresis gels. The gels were transferred to a polyvinylidene fluoride (PVDF) membrane at 300 mA for 2.5 h at 4°C. Subsequently, the membranes were blocked with 5% bovine serum albumin (BSA) for 1 h at room temperature (RT), followed by three washes with 0.1% Tween-20 in TBS (TBST). The membranes were incubated with primary Abs diluted at 1:500 in TBST with 1% BSA overnight at 4°C. After three washes with TBST, the blots were incubated with the HRP-labeled secondary goat anti-rabbit or donkey anti-goat Ab respectively for 1 h at RT. After three washes, the blots were imaged [81].

### Immunofluorescent staining

The collected cells were fixed in 4% paraformaldehyde for 1 h, then the cells were spread onto poly-L-lysine coated microscope slides and air-dry. After three washings with PBS (5 min each) cells were incubated with 2% (v/v) Triton X-100 in PBS for 1 h at RT. Then, after three washes with PBS, the cells were blocked with 1% (wt/v) BSA and 1% goat serum in PBS for 30 min at RT, then incubation with primary antibodies diluted in blocking solution overnight at 4°C. The information for the primary antibodies (Abs) is present in Supplementary Table 2. The following morning, after three washes with PBS Tween 20 (0.5%) the slides were incubated with Alexa Fluor 546 goat anti-rabbit IgG (1:200) for 30 min in darkness at RT. The negative controls samples were incubated with secondary antibody and without primary antibody. Slides were washed with PBS Tween-20 three times and then incubated with DAPI (4.6-diamidino-2-phenylindole hydrochloride, 100 ng/ml) as nuclear stain for 5 min. After brief wash with ddH_2_O, the slides were covered with an anti-fading mounting medium (Vector, Burlingame, USA). Fluorescent images were obtained with Leica Laser Scanning Confocal Microscope (LEICA TCS SP5 II, Germany) [118].

### Statistical analyses

The data were statistically analyzed using SPSS statistical software (IBM Co., NY, USA) and ANOVA. Comparisons between groups were tested by One-Way ANOVA analysis and the LSD test. All the groups were compared with each other for every parameter (mean ± SE). Differences were considered significant at *P* < 0.05.

## SUPPLEMENTARY FIGURES AND TABLES



## Abbreviations

Ag: silver
ATCC: American Type Culture Collection
ATM: Ataxia telangiectasia mutated
ATR: ATM- and Rad3-related
Au: gold
CuO: cupric oxide
DAPI: 4′,6-diamidino-2-phenylindole
DLS: dynamic light scattering
DMEM: Dulbecco's modified Eagle's medium
DNA-PK: DNA-dependent protein kinase
DSB: double strand break
EDS: Energy Disperse Spectroscopy
EdU: 5-Ethynyl-2′-deoxyuridine
FBS: Fetal bovine serum
GAPDH: glyceraldehyde-3-phosphate dehydrogenase
HRP: horse radish peroxidase
IKK: IκB kinase
LPS: lipopolysaccharide
MTT: 3-(4, 5)-dimethylthiazol-2, 5-diphenyltetrazolium bromide
NaCl: sodium chloride
ND: diamond nanoparticle
NF-κB: Nuclear factor kappa-light-chain-enhancer of activated B
NPs: nanoparticles
PCNA: Proliferating Cell Nuclear Antigen
PP2A: Protein phosphatase 2A
PP2AC: Protein phosphatase2A homologues
PP4C: Protein phosphatase 4 catalytic subunit
PP4R2: Protein phosphatase 4 regulator subunit 2
PP4R3: Protein phosphatase 4 regulatory subunit 3 beta
PP6C: Protein phosphatase 6 catalytic subunit
Pt: platinium
PVDF: polyvinylidene fluoride
ROS: reactive oxygen species
RT: room temperature
SiO_2_: silicon dioxide
TBS: Tris buffer saline
TBST: Tris buffer saline Tween 20
TEM: transmission electron microscopy
TiO_2_: titanium dioxide
TUNEL: Terminal deoxynucleotidyl transferase dUTP nick end labeling
VYS: visceral yolk sac
ZnO: Zinc oxide
ZnSO_4_: Zinc sulfide

## References

[1] Austin CA, Hinkley GK, Mishra AR, Zhang Q, Umbreit TH, Betz MW, E Wildt B, Casey BJ, Francke-Carroll S, Hussain SM, Roberts SM, Brown KM, Goering PL (2016). Distribution and accumulation of 10 nm silver nanoparticles in maternal tissues and visceral yolk sac of pregnant mice, and a potential effect on embryo growth. Nanotoxicology.

[2] Kalishwaralal K, Jeyabharathi S, Sundar K, Muthukumaran A (2016). A novel one-pot green synthesis of selenium nanoparticles and evaluation of its toxicity in zebrafish embryos. Artif Cells Nanomed Biotechnol.

[3] Lacave JM, Retuerto A, Vicario-Parés U, Gilliland D, Oron M, Cajaraville MP, Orbea A (2016). Effects of metal-bearing nanoparticles (Ag, Au, CdS, ZnO, SiO2) on developing zebrafish embryos. Nanotechnology.

[4] Wang Y, Zhou J, Liu L, Huang C, Zhou D, Fu L (2016). Characterization and toxicology evaluation of chitosan nanoparticles on the embryonic development of zebrafish, Danio rerio. Carbohydr Polym.

[5] Yan D, Ni LK, Chen HL, Chen LC, Chen YH, Cheng CC (2016). Amphiphilic nanoparticles of resveratrol-norcantharidin to enhance the toxicity in zebrafish embryo. Bioorg Med Chem Lett.

[6] Ahmad F, Liu X, Zhou Y, Yao H (2015). An in vivo evaluation of acute toxicity of cobalt ferrite (CoFe2O4) nanoparticles in larval-embryo Zebrafish (Danio rerio). Aquat Toxicol.

[7] Bonfanti P, Moschini E, Saibene M, Bacchetta R, Rettighieri L, Calabri L, Colombo A, Mantecca P (2015). Do Nanoparticle Physico-Chemical Properties and Developmental Exposure Window Influence Nano ZnO Embryotoxicity in Xenopus laevis?. Int J Environ Res Public Health.

[8] Groh KJ, Dalkvist T, Piccapietra F, Behra R, Suter MJ, Schirmer K (2015). Critical influence of chloride ions on silver ion-mediated acute toxicity of silver nanoparticles to zebrafish embryos. Nanotoxicology.

[9] Parivar K, Hayati Rudbari N, Khanbabaee R, Khaleghi M (2015). The Effect of Nano-Titanium Dioxide on Limb Bud Development of NMRI Mouse Embryo In Vivo. Cell J.

[10] Ruyra À, Yazdi A, Espín J, Carné-Sánchez A, Roher N, Lorenzo J, Imaz I, Maspoch D (2015). Synthesis, culture medium stability, and in vitro and in vivo zebrafish embryo toxicity of metal-organic framework nanoparticles. Chemistry.

[11] Wang ZG, Zhou R, Jiang D, Song JE, Xu Q, Si J, Chen YP, Zhou X, Gan L, Li JZ, Zhang H, Liu B (2015). Toxicity of Graphene Quantum Dots in Zebrafish Embryo. Biomed Environ Sci.

[12] Yoisungnern T, Choi YJ, Han JW, Kang MH, Das J, Gurunathan S, Kwon DN, Cho SG, Park C, Chang WK, Chang BS, Parnpai R, Kim JH (2015). Internalization of silver nanoparticles into mouse spermatozoa results in poor fertilization and compromised embryo development. Sci Rep.

[13] Zhang XF, Park JH, Choi YJ, Kang MH, Gurunathan S, Kim JH (2015). Silver nanoparticles cause complications in pregnant mice. Int J Nanomedicine.

[14] Brun NR, Lenz M, Wehrli B, Fent K (2014). Comparative effects of zinc oxide nanoparticles and dissolved zinc on zebrafish embryos and eleuthero-embryos: importance of zinc ions. Sci Total Environ.

[15] Chen TH, Lin CC, Meng PJ (2014). Zinc oxide nanoparticles alter hatching and larval locomotor activity in zebrafish (Danio rerio). J Hazard Mater.

[16] George S, Gardner H, Seng EK, Chang H, Wang C, Yu Fang CH, Richards M, Valiyaveettil S, Chan WK (2014). Differential effect of solar light in increasing the toxicity of silver and titanium dioxide nanoparticles to a fish cell line and zebrafish embryos. Environ Sci Technol.

[17] He X, Aker WG, Hwang HM (2014). An in vivo study on the photo-enhanced toxicities of S-doped TiO2 nanoparticles to zebrafish embryos (Danio rerio) in terms of malformation, mortality, rheotaxis dysfunction, and DNA damage. Nanotoxicology.

[18] Hong JS, Park MK, Kim MS, Lim JH, Park GJ, Maeng EH, Shin JH, Kim MK, Jeong J, Park JA, Kim JC, Shin HC (2014). Prenatal development toxicity study of zinc oxide nanoparticles in rats. Int J Nanomedicine.

[19] Hong JS, Park MK, Kim MS, Lim JH, Park GJ, Maeng EH, Shin JH, Kim YR, Kim MK, Lee JK, Park JA, Kim JC, Shin HC (2014). Effect of zinc oxide nanoparticles on dams and embryo-fetal development in rats. Int J Nanomedicine.

[20] Hua J, Vijver MG, Richardson MK, Ahmad F, Peijnenburg WJ (2014). Particle-specific toxic effects of differently shaped zinc oxide nanoparticles to zebrafish embryos (Danio rerio). Environ Toxicol Chem.

[21] Kim MS, Louis KM, Pedersen JA, Hamers RJ, Peterson RE, Heideman W (2014). Using citrate-functionalized TiO2 nanoparticles to study the effect of particle size on zebrafish embryo toxicity. Analyst (Lond).

[22] Kim KT, Tanguay RL (2014). The role of chorion on toxicity of silver nanoparticles in the embryonic zebrafish assay. Environ Health Toxicol.

[23] Lee BC, Kim J, Cho JG, Lee JW, Duong CN, Bae E, Yi J, Eom IC, Choi K, Kim P, Yoon J (2014). Effects of ionization on the toxicity of silver nanoparticles to Japanese medaka (Oryzias latipes) embryos. J Environ Sci Health A Tox Hazard Subst Environ Eng.

[24] Massarsky A, Strek L, Craig PM, Eisa-Beygi S, Trudeau VL, Moon TW (2014). Acute embryonic exposure to nanosilver or silver ion does not disrupt the stress response in zebrafish (Danio rerio) larvae and adults. Sci Total Environ.

[25] McNeil PL, Boyle D, Henry TB, Handy RD, Sloman KA (2014). Effects of metal nanoparticles on the lateral line system and behaviour in early life stages of zebrafish (Danio rerio). Aquat Toxicol.

[26] Ong KJ, Zhao X, Thistle ME, Maccormack TJ, Clark RJ, Ma G, Martinez-Rubi Y, Simard B, Loo JS, Veinot JG, Goss GG (2014). Mechanistic insights into the effect of nanoparticles on zebrafish hatch. Nanotoxicology.

[27] Ozel RE, Wallace KN, Andreescu S (2014). Alterations of intestinal serotonin following nanoparticle exposure in embryonic zebrafish. Environ Sci Nano.

[28] Pavagadhi S, Sathishkumar M, Balasubramanian R (2014). Uptake of Ag and TiO2 nanoparticles by zebrafish embryos in the presence of other contaminants in the aquatic environment. Water Res.

[29] Shin YJ, Nam SH, An YJ (2014). Japanese medaka exposed to gold nanoparticles: only embryonic exposure generates irreversible hatching failure, developmental failure, and mortality of sac-fry. Comp Biochem Physiol C Toxicol Pharmacol.

[30] Tsyganova NA, Khairullin RM, Terentyuk GS, Khlebtsov BN, Bogatyrev VA, Dykman LA, Erykov SN, Khlebtsov NG (2014). Penetration of pegylated gold nanoparticles through rat placental barrier. Bull Exp Biol Med.

[31] Yan J, Lin B, Hu C, Zhang H, Lin Z, Xi Z (2014). The combined toxicological effects of titanium dioxide nanoparticles and bisphenol A on zebrafish embryos. Nanoscale Res Lett.

[32] Yu WJ, Son JM, Lee J, Kim SH, Lee IC, Baek HS, Shin IS, Moon C, Kim SH, Kim JC (2014). Effects of silver nanoparticles on pregnant dams and embryo-fetal development in rats.

[33] Browning LM, Lee KJ, Nallathamby PD, Xu XH (2013). Silver nanoparticles incite size- and dose-dependent developmental phenotypes and nanotoxicity in zebrafish embryos. Chem Res Toxicol.

[34] Cho JG, Kim KT, Ryu TK, Lee JW, Kim JE, Kim J, Lee BC, Jo EH, Yoon J, Eom IC, Choi K, Kim P (2013). Stepwise embryonic toxicity of silver nanoparticles on Oryzias latipes. Biomed Res Int.

[35] Duan J, Yu Y, Shi H, Tian L, Guo C, Huang P, Zhou X, Peng S, Sun Z (2013). Toxic effects of silica nanoparticles on zebrafish embryos and larvae. PLoS One.

[36] Grodzik M, Sawosz F, Sawosz E, Hotowy A, Wierzbicki M, Kutwin M, Jaworski S, Chwalibog A (2013). Nano-nutrition of chicken embryos. The effect of in ovo administration of diamond nanoparticles and L-glutamine on molecular responses in chicken embryo pectoral muscles. Int J Mol Sci.

[37] Kim KT, Truong L, Wehmas L, Tanguay RL (2013). Silver nanoparticle toxicity in the embryonic zebrafish is governed by particle dispersion and ionic environment. Nanotechnology.

[38] Lee KJ, Browning LM, Nallathamby PD, Osgood CJ, Xu XH (2013). Silver nanoparticles induce developmental stage-specific embryonic phenotypes in zebrafish. Nanoscale.

[39] Libralato G, Minetto D, Totaro S, Mičetić I, Pigozzo A, Sabbioni E, Marcomini A, Volpi Ghirardini A (2013). Embryotoxicity of TiO2 nanoparticles to Mytilus galloprovincialis (Lmk). Mar Environ Res.

[40] Manzo S, Miglietta ML, Rametta G, Buono S, Di Francia G (2013). Embryotoxicity and spermiotoxicity of nanosized ZnO for Mediterranean sea urchin Paracentrotus lividus. J Hazard Mater.

[41] Muth-Köhne E, Sonnack L, Schlich K, Hischen F, Baumgartner W, Hund-Rinke K, Schäfers C, Fenske M (2013). The toxicity of silver nanoparticles to zebrafish embryos increases through sewage treatment processes. Ecotoxicology.

[42] Park MR, Gurunathan S, Choi YJ, Kwon DN, Han JW, Cho SG, Park C, Seo HG, Kim JH (2013). Chitosan nanoparticles cause pre- and postimplantation embryo complications in mice. Biol Reprod.

[43] Park K, Tuttle G, Sinche F, Harper SL (2013). Stability of citrate-capped silver nanoparticles in exposure media and their effects on the development of embryonic zebrafish (Danio rerio). Arch Pharm Res.

[44] Prasek M, Sawosz E, Jaworski S, Grodzik M, Ostaszewska T, Kamaszewski M, Wierzbicki M, Chwalibog A (2013). Influence of nanoparticles of platinum on chicken embryo development and brain morphology. Nanoscale Res Lett.

[45] Rizzo LY, Golombek SK, Mertens ME, Pan Y, Laaf D, Broda J, Jayapaul J, Möckel D, Subr V, Hennink WE, Storm G, Simon U, Jahnen-Dechent W (2013). In Vivo Nanotoxicity Testing using the Zebrafish Embryo Assay. J Mater Chem B Mater Biol Med.

[46] Truong L, Tilton SC, Zaikova T, Richman E, Waters KM, Hutchison JE, Tanguay RL (2013). Surface functionalities of gold nanoparticles impact embryonic gene expression responses. Nanotoxicology.

[47] Zhao X, Wang S, Wu Y, You H, Lv L (2013). Acute ZnO nanoparticles exposure induces developmental toxicity, oxidative stress and DNA damage in embryo-larval zebrafish. Aquat Toxicol.

[48] Austin CA, Umbreit TH, Brown KM, Barber DS, Dair BJ, Francke-Carroll S, Feswick A, Saint-Louis MA, Hikawa H, Siebein KN, Goering PL (2012). Distribution of silver nanoparticles in pregnant mice and developing embryos. Nanotoxicology.

[49] Lee KJ, Browning LM, Nallathamby PD, Desai T, Cherukuri PK, Xu XH (2012). In vivo quantitative study of sized-dependent transport and toxicity of single silver nanoparticles using zebrafish embryos. Chem Res Toxicol.

[50] Truong L, Saili KS, Miller JM, Hutchison JE, Tanguay RL (2012). Persistent adult zebrafish behavioral deficits results from acute embryonic exposure to gold nanoparticles. Comp Biochem Physiol C Toxicol Pharmacol.

[51] Truong L, Zaikova T, Richman EK, Hutchison JE, Tanguay RL (2012). Media ionic strength impacts embryonic responses to engineered nanoparticle exposure. Nanotoxicology.

[52] Yang H, Sun C, Fan Z, Tian X, Yan L, Du L, Liu Y, Chen C, Liang XJ, Anderson GJ, Keelan JA, Zhao Y, Nie G (2012). Effects of gestational age and surface modification on materno-fetal transfer of nanoparticles in murine pregnancy. Sci Rep.

[53] Zhang W, Lin K, Sun X, Dong Q, Huang C, Wang H, Guo M, Cui X (2012). Toxicological effect of MPA-CdSe QDs exposure on zebrafish embryo and larvae. Chemosphere.

[54] Zhu X, Tian S, Cai Z (2012). Toxicity assessment of iron oxide nanoparticles in zebrafish (Danio rerio) early life stages. PLoS One.

[55] Asharani PV, Lianwu Y, Gong Z, Valiyaveettil S (2011). Comparison of the toxicity of silver, gold and platinum nanoparticles in developing zebrafish embryos. Nanotoxicology.

[56] Cowart DA, Guida SM, Shah SI, Marsh AG (2011). Effects of Ag nanoparticles on survival and oxygen consumption of zebra fish embryos, Danio rerio. J Environ Sci Health A Tox Hazard Subst Environ Eng.

[57] Hu YL, Qi W, Han F, Shao JZ, Gao JQ (2011). Toxicity evaluation of biodegradable chitosan nanoparticles using a zebrafish embryo model. Int J Nanomedicine.

[58] Lee WM, Ha SW, Yang CY, Lee JK, An YJ (2011). Effect of fluorescent silica nanoparticles in embryo and larva of Oryzias latipes: sonic effect in nanoparticle dispersion. Chemosphere.

[59] Truong L, Moody IS, Stankus DP, Nason JA, Lonergan MC, Tanguay RL (2011). Differential stability of lead sulfide nanoparticles influences biological responses in embryonic zebrafish. Arch Toxicol.

[60] Umanzor-Alvarez J, Wade EC, Gifford A, Nontapot K, Cruz-Reese A, Gotoh T, Sible JC, Khodaparast GA (2011). Near-infrared laser delivery of nanoparticles to developing embryos: a study of efficacy and viability. Biotechnol J.

[61] Verma S, Das S, Khanagrot BS (2011). Effects of CdO nanoparticles on the development and hatching of a freshwater pulmonate snail Lymnaea luteola L. J Biomed Nanotechnol.

[62] Xia T, Zhao Y, Sager T, George S, Pokhrel S, Li N, Schoenfeld D, Meng H, Lin S, Wang X, Wang M, Ji Z, Zink JI (2011). Decreased dissolution of ZnO by iron doping yields nanoparticles with reduced toxicity in the rodent lung and zebrafish embryos. ACS Nano.

[63] Yamashita K, Yoshioka Y, Higashisaka K, Mimura K, Morishita Y, Nozaki M, Yoshida T, Ogura T, Nabeshi H, Nagano K, Abe Y, Kamada H, Monobe Y (2011). Silica and titanium dioxide nanoparticles cause pregnancy complications in mice. Nat Nanotechnol.

[64] Bai W, Tian W, Zhang Z, He X, Ma Y, Liu N, Chai Z (2010). Effects of copper nanoparticles on the development of zebrafish embryos. J Nanosci Nanotechnol.

[65] Ringwood AH, McCarthy M, Bates TC, Carroll DL (2010). The effects of silver nanoparticles on oyster embryos. Mar Environ Res.

[66] Bar-Ilan O, Albrecht RM, Fako VE, Furgeson DY (2009). Toxicity assessments of multisized gold and silver nanoparticles in zebrafish embryos. Small.

[67] Browning LM, Lee KJ, Huang T, Nallathamby PD, Lowman JE, Xu XH (2009). Random walk of single gold nanoparticles in zebrafish embryos leading to stochastic toxic effects on embryonic developments. Nanoscale.

[68] King-Heiden TC, Wiecinski PN, Mangham AN, Metz KM, Nesbit D, Pedersen JA, Hamers RJ, Heideman W, Peterson RE (2009). Quantum dot nanotoxicity assessment using the zebrafish embryo. Environ Sci Technol.

[69] Zhu X, Zhu L, Duan Z, Qi R, Li Y, Lang Y (2008). Comparative toxicity of several metal oxide nanoparticle aqueous suspensions to Zebrafish (Danio rerio) early developmental stage. J Environ Sci Health A Tox Hazard Subst Environ Eng.

[70] Lee KJ, Nallathamby PD, Browning LM, Osgood CJ, Xu XH (2007). In vivo imaging of transport and biocompatibility of single silver nanoparticles in early development of zebrafish embryos. ACS Nano.

[71] Bosman SJ, Nieto SP, Patton WC, Jacobson JD, Corselli JU, Chan PJ (2005). Development of mammalian embryos exposed to mixed-size nanoparticles. Clin Exp Obstet Gynecol.

[72] Bondarenko O, Juganson K, Ivask A, Kasemets K, Mortimer M, Kahru A (2013). Toxicity of Ag, CuO and ZnO nanoparticles to selected environmentally relevant test organisms and mammalian cells in vitro: a critical review. Arch Toxicol.

[73] Ma H, Williams PL, Diamond SA (2013). Ecotoxicity of manufactured ZnO nanoparticles—a review. Environ Pollut.

[74] Cho WS, Kang BC, Lee JK, Jeong J, Che JH, Seok SH (2013). Comparative absorption, distribution, and excretion of titanium dioxide and zinc oxide nanoparticles after repeated oral administration. Part Fibre Toxicol.

[75] Lopes S, Ribeiro F, Wojnarowicz J, Łojkowski W, Jurkschat K, Crossley A, Soares AM, Loureiro S (2014). Zinc oxide nanoparticles toxicity to Daphnia magna: size-dependent effects and dissolution. Environ Toxicol Chem.

[76] Talebi AR, Khorsandi L, Moridian M (2013). The effect of zinc oxide nanoparticles on mouse spermatogenesis. J Assist Reprod Genet.

[77] Jo E, Seo G, Kwon JT, Lee M, Lee B, Eom I, Kim P, Choi K (2013). Exposure to zinc oxide nanoparticles affects reproductive development and biodistribution in offspring rats. J Toxicol Sci.

[78] Zhao Y, Li L, Zhang PF, Liu XQ, Zhang WD, Ding ZP, Wang SW, Shen W, Min LJ, Hao ZH (2016). Regulation of egg quality and lipids metabolism by Zinc Oxide Nanoparticles. Poult Sci.

[79] Zhao Y, Li L, Zhang PF, Shen W, Liu J, Yang FF, Liu HB, Hao ZH (2015). Differential regulation of gene and protein expression by zinc oxide nanoparticles in hen's ovarian granulosa cells: specific roles of nanoparticles. PLoS One.

[80] Mahalingaiah S, Hart JE, Laden F, Farland LV, Hewlett MM, Chavarro J, Aschengrau A, Missmer SA (2016). Adult air pollution exposure and risk of infertility in the Nurses’ Health Study II. Hum Reprod.

[81] Samet JM, DeMarini DM, Malling HV (2004). Biomedicine. Do airborne particles induce heritable mutations?. Science.

[82] Pal A, Alam S, Mittal S, Arjaria N, Shankar J, Kumar M, Singh D, Pandey AK, Ansari KM (2016). UVB irradiation-enhanced zinc oxide nanoparticles-induced DNA damage and cell death in mouse skin. Mutat Res Genet Toxicol Environ Mutagen.

[83] Prasad RY, Chastain PD, Nikolaishvili-Feinberg N, Smeester L, Kaufmann WK, Fry RC (2013). Titanium dioxide nanoparticles activate the ATM-Chk2 DNA damage response in human dermal fibroblasts. Nanotoxicology.

[84] Wan R, Mo Y, Feng L, Chien S, Tollerud DJ, Zhang Q (2012). DNA damage caused by metal nanoparticles: involvement of oxidative stress and activation of ATM. Chem Res Toxicol.

[85] Condello M, De Berardis B, Ammendolia MG, Barone F, Condello G, Degan P, Meschini S (2016). ZnO nanoparticle tracking from uptake to genotoxic damage in human colon carcinoma cells. Toxicol In Vitro.

[86] Turinetto V, Giachino C (2015). Multiple facets of histone variant H2AX: a DNA double-strand-break marker with several biological functions. Nucleic Acids Res.

[87] Xie A, Puget N, Shim I, Odate S, Jarzyna I, Bassing CH, Alt FW, Scully R (2004). Control of sister chromatid recombination by histone H2AX. Mol Cell.

[88] Kafer GR, Lehnert SA, Pantaleon M, Kaye PL, Moser RJ (2010). Expression of genes coding for histone variants and histone-associated proteins in pluripotent stem cells and mouse preimplantation embryos. Gene Expr Patterns.

[89] Nashun B, Yukawa M, Liu H, Akiyama T, Aoki F (2010). Changes in the nuclear deposition of histone H2A variants during pre-implantation development in mice. Development.

[90] Turinetto V, Orlando L, Sanchez-Ripoll Y, Kumpfmueller B, Storm MP, Porcedda P, Minieri V, Saviozzi S, Accomasso L, Cibrario Rocchietti E, Moorwood K, Circosta P, Cignetti A (2012). High basal γH2AX levels sustain self-renewal of mouse embryonic and induced pluripotent stem cells. Stem Cells.

[91] Rankin EB, Giaccia AJ, Hammond EM (2009). Bringing H2AX into the angiogenesis family. Cancer Cell.

[92] Economopoulou M, Langer HF, Celeste A, Orlova VV, Choi EY, Ma M, Vassilopoulos A, Callen E, Deng C, Bassing CH, Boehm M, Nussenzweig A, Chavakis T (2009). Histone H2AX is integral to hypoxia-driven neovascularization. Nat Med.

[93] Hammond EM, Dorie MJ, Giaccia AJ (2003). ATR/ATM targets are phosphorylated by ATR in response to hypoxia and ATM in response to reoxygenation. J Biol Chem.

[94] Goldstein S (1990). Replicative senescence: the human fibroblast comes of age. Science.

[95] Kuilman T, Peeper DS (2009). Senescence-messaging secretome: SMS-ing cellular stress. Nat Rev Cancer.

[96] Campisi J, d’Adda di Fagagna F (2007). Cellular senescence: when bad things happen to good cells. Nat Rev Mol Cell Biol.

[97] Chowdhury D, Keogh MC, Ishii H, Peterson CL, Buratowski S, Lieberman J (2005). gamma-H2AX dephosphorylation by protein phosphatase 2A facilitates DNA double-strand break repair. Mol Cell.

[98] Chowdhury D, Xu X, Zhong X, Ahmed F, Zhong J, Liao J, Dykxhoorn DM, Weinstock DM, Pfeifer GP, Lieberman J (2008). A PP4-phosphatase complex dephosphorylates gamma-H2AX generated during DNA replication. Mol Cell.

[99] Honkanen RE, Golden T (2002). Regulators of serine/threonine protein phosphatases at the dawn of a clinical era?. Curr Med Chem.

[100] Keogh MC, Kim JA, Downey M, Fillingham J, Chowdhury D, Harrison JC, Onishi M, Datta N, Galicia S, Emili A, Lieberman J, Shen X, Buratowski S (2006). A phosphatase complex that dephosphorylates gammaH2AX regulates DNA damage checkpoint recovery. Nature.

[101] Mukherjee SP, Quintas PO, McNulty R, Komives EA, Dyson HJ (2016). Structural characterization of the ternary complex that mediates termination of NF-κB signaling by IκBα. Proc Natl Acad Sci U S A.

[102] Harhaj EW, Dixit VM (2011). Deubiquitinases in the regulation of NF-κB signaling. Cell Res.

[103] Shembade N, Harhaj EW (2012). Regulation of NF-κB signaling by the A20 deubiquitinase. Cell Mol Immunol.

[104] Bernal GM, Wahlstrom JS, Crawley CD, Cahill KE, Pytel P, Liang H, Kang S, Weichselbaum RR, Yamini B (2014). Loss of Nfkb1 leads to early onset aging. Aging (Albany NY).

[105] Vyas D, Lopez-Hisijos N, Gandhi S, El-Dakdouki M, Basson MD, Walsh MF, Huang X, Vyas AK, Chaturvedi LS (2015). Doxorubicin-Hyaluronan Conjugated Super-Paramagnetic Iron Oxide Nanoparticles (DOX-HA-SPION) Enhanced Cytoplasmic Uptake of Doxorubicin and Modulated Apoptosis, IL-6 Release and NF-kappaB Activity in Human MDA-MB-231 Breast Cancer Cells. J Nanosci Nanotechnol.

[106] Sarkar S, Leo BF, Carranza C, Chen S, Rivas-Santiago C, Porter AE, Ryan MP, Gow A, Chung KF, Tetley TD, Zhang JJ, Georgopoulos PG, Ohman-Strickland PA (2015). Modulation of Human Macrophage Responses to Mycobacterium tuberculosis by Silver Nanoparticles of Different Size and Surface Modification. PLoS One.

[107] Opipari AW, Boguski MS, Dixit VM (1990). The A20 cDNA induced by tumor necrosis factor alpha encodes a novel type of zinc finger protein. J Biol Chem.

[108] Opipari AW, Hu HM, Yabkowitz R, Dixit VM (1992). The A20 zinc finger protein protects cells from tumor necrosis factor cytotoxicity. J Biol Chem.

[109] Song HY, Rothe M, Goeddel DV (1996). The tumor necrosis factor-inducible zinc finger protein A20 interacts with TRAF1/TRAF2 and inhibits NF-kappaB activation. Proc Natl Acad Sci USA.

[110] Heyninck K, De Valck D, Vanden Berghe W, Van Criekinge W, Contreras R, Fiers W, Haegeman G, Beyaert R (1999). The zinc finger protein A20 inhibits TNF-induced NF-kappaB-dependent gene expression by interfering with an RIP- or TRAF2-mediated transactivation signal and directly binds to a novel NF-kappaB-inhibiting protein ABIN. J Cell Biol.

[111] Jäättelä M, Mouritzen H, Elling F, Bastholm L (1996). A20 zinc finger protein inhibits TNF and IL-1 signaling. J Immunol.

[112] Kim MH, Jeong HJ (2015). Zinc Oxide Nanoparticles Suppress LPS-Induced NF-κB Activation by Inducing A20, a Negative Regulator of NF-κB, in RAW 264.7 Macrophages. J Nanosci Nanotechnol.

[113] Feng H, Pyykkö I, Zou J (2016). Involvement of Ubiquitin-Editing Protein A20 in Modulating Inflammation in Rat Cochlea Associated with Silver Nanoparticle-Induced CD68 Upregulation and TLR4 Activation. Nanoscale Res Lett.

[114] James SA, Feltis BN, de Jonge MD, Sridhar M, Kimpton JA, Altissimo M, Mayo S, Zheng C, Hastings A, Howard DL, Paterson DJ, Wright PF, Moorhead GF (2013). Quantification of ZnO nanoparticle uptake, distribution, and dissolution within individual human macrophages. ACS Nano.

[115] García-Hevia L, Valiente R, Martín-Rodríguez R, Renero-Lecuna C, González J, Rodríguez-Fernández L, Aguado F, Villegas JC, Fanarraga ML (2016). Nano-ZnO leads to tubulin macrotube assembly and actin bundling, triggering cytoskeletal catastrophe and cell necrosis. Nanoscale.

[116] Xia T, Kovochich M, Liong M, Mädler L, Gilbert B, Shi H, Yeh JI, Zink JI, Nel AE (2008). Comparison of the mechanism of toxicity of zinc oxide and cerium oxide nanoparticles based on dissolution and oxidative stress properties. ACS Nano.

[117] Zhao Y, Li L, Min LJ, Zhu LQ, Sun QY, Zhang HF, Liu XQ, Zhang WD, Ge W, Wang JJ, Liu JC, Hao ZH (2016). Regulation of MicroRNAs, and the Correlations of MicroRNAs and Their Targeted Genes by Zinc Oxide Nanoparticles in Ovarian Granulosa Cells. PLoS One.

[118] Liu XQ, Zhang HF, Zhang WD, Zhang PF, Hao YN, Song R, Li L, Feng YN, Hao ZH, Shen W, Min LJ, Yang HD, Zhao Y (2016). Regulation of neuroendocrine cells and neuron factors in the ovary by zinc oxide nanoparticles. Toxicol Lett.

[119] Zhang WD, Zhao Y, Zhang HF, Wang SK, Hao ZH, Liu J, Yuan YQ, Zhang PF, Yang HD, Shen W, Li L (2016). Alteration of gene expression by zinc oxide nanoparticles or zinc sulfate in vivo and comparison with in vitro data: A harmonious case. Theriogenology.

[120] Siopes TD (1999). Improved effectiveness of artificial insemination of turkey hens associated with ahemeral light-dark cycles and age at photostimulation. Poult Sci.

[121] É Váradi, Végi B, Liptói K, Barna J (2013). Methods for cryopreservation of guinea fowl sperm. PLoS One.

[122] Liptói K, Varga A, Hidas A, Barna J (2004). Determination of the rate of true fertility in duck breeds by the combination of two in vitro methods. Acta Vet Hung.

[123] Yang M, Zhang C, Zhang X, Zhang MZ, Rottinghaus GE, Zhang S (2016). Structure-function analysis of Avian β-defensin-6 and β-defensin-12: role of charge and disulfide bridges. BMC Microbiol.

[124] Xiao J, Lin HY, Zhu YY, Zhu YP, Chen LW (2016). MiR-126 regulates proliferation and invasion in the bladder cancer BLS cell line by targeting the PIK3R2-mediated PI3K/Akt signaling pathway. Onco Targets Ther.

