# Supplementary material for: Oocyte exposure to ZnO nanoparticles inhibits early embryonic development through the γ-H2AX and NF-κB signaling pathways
Source: Oncotarget. 2017 Apr 21;8(26):42673–92. doi: 10.18632/oncotarget.17349 (PMC5522097; doi:10.18632/oncotarget.17349)
Supplement: Supplementary file 1 [file oncotarget-08-42673-s001.pdf]

Oocyte exposure to ZnO nanoparticles inhibits early embryonic development through the γ-H2AX and NF-κB signaling pathways

Supplementary Materials

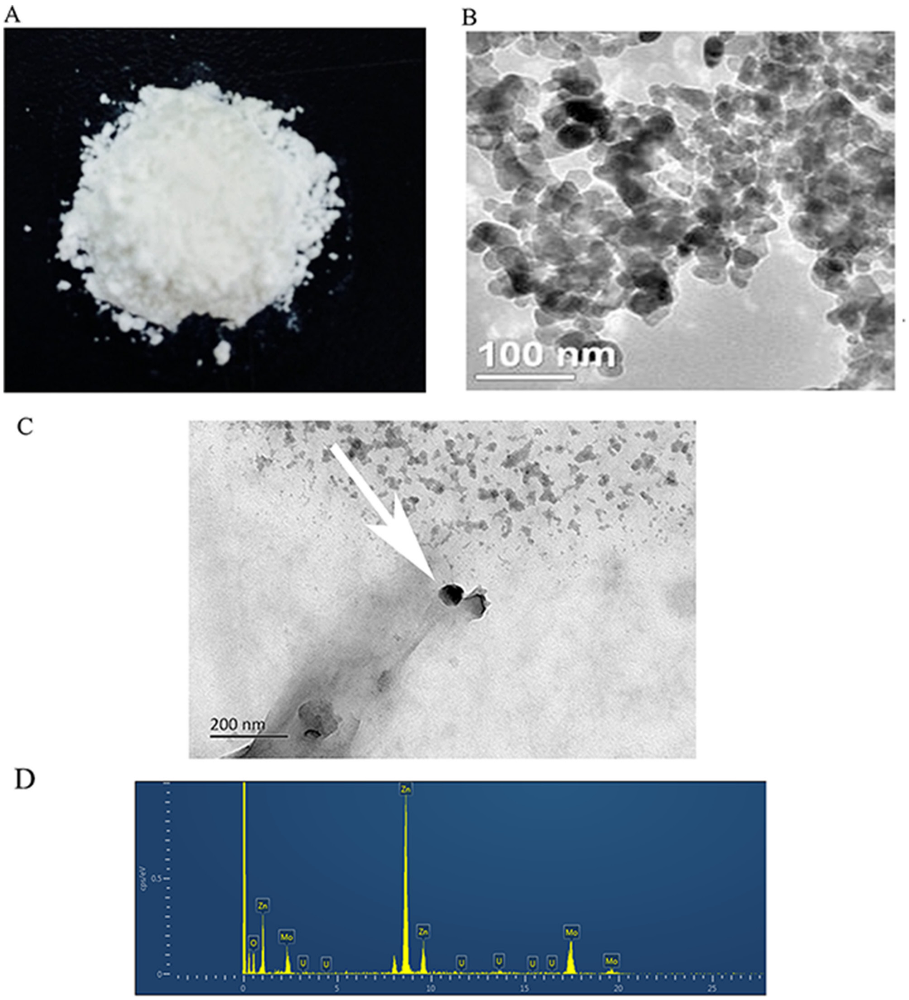

Supplementary Figure 1: ZnO NPs in ovary. (**A**) Photo of ZnO NPs. (**B**) TEM photo of ZnO NPs and the characteristics of them. (**C**) TEM photo of ZnO NPs in ovary indicated by the white arrow. D. EDS picture of ZnO NPs in ovary, where three Zn peaks have shown [81].

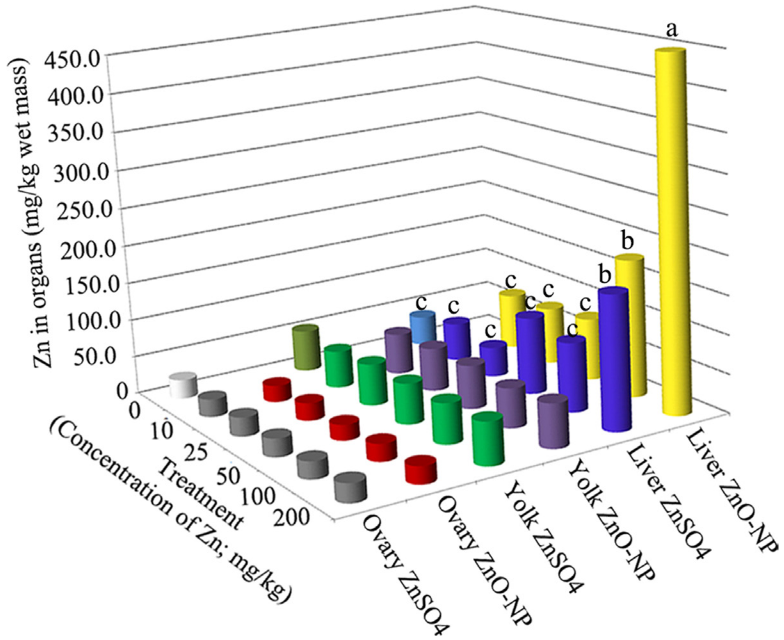

Supplementary Figure 2: Effects of ZnO NPs and ZnSO4 treatments on Zn contents in liver, ovary and yolk. Z axis is the content (mg/kg wet mass), and Y axis is the treatment (concentration of Zn; mg/kg), X axis shows the treatment groups. [a-c] Means for Zn content not sharing a common superscript are different ($P < 0.05$) [80].

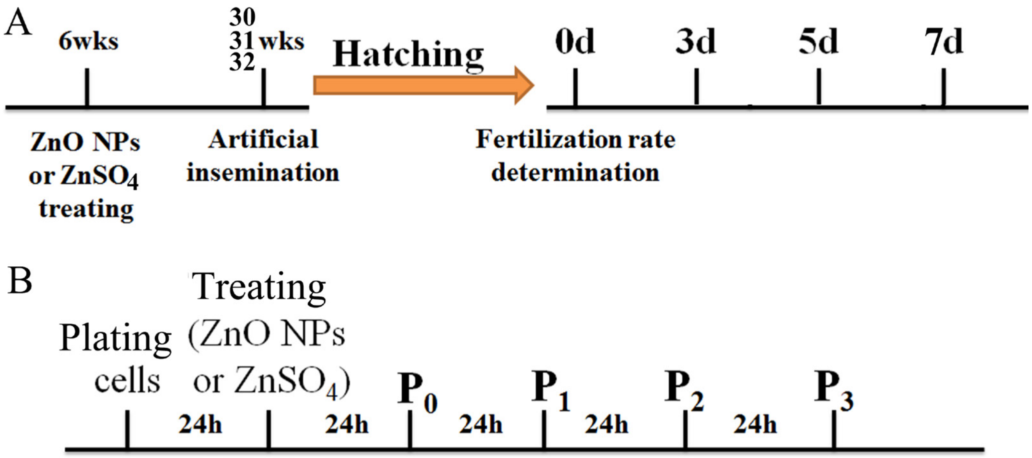

Supplementary Figure 3: Time schemes for the study design. (**A**) Time scheme for animal study; (**B**) Time scheme for *in vitro* culture study.

Supplementary Table 1: Ingredient composition of the basal diet for the layers

Item	Amount (6–18 wks of age)	Amount (19–30 wks of age)
**Ingredient**		
Corn, %	66.10	62.10
Soybean meal, %	20.00	24.00
Limestone, %	8.00	8.00
Vitamin-mineral premix[1]	5.00	5.00
Soybean oil	0.90	0.90
Total	100.00	100.00
**Nutrient level**		
Metabolizable energy, (MJ/kg)	11.27	11.58
CP (%)	14.12 (14.25)[2]	15.98 (16.12)[2]
Calcium (%)	3.21 (3.17)[2]	3.11 (3.05)[2]
Nonphytate phosphorus (%)	0.33	0.31
Lysine (%)	0.69	0.72
Methionine (%)	0.31	0.36
Methionine + cysteine (%)	0.62	0.66
Zn (ppm)	11.98	12.03

[1]Provided per kilogram of product: 181,000 IU of vitamin A; 37,000 IU of vitamin D_3; 510 mg of vitamin E; 35 mg of vitamin K_3; 33.5 mg vitamin B1; 72 mg vitamin B2; 16 mg vitamin B6; 0.5 mg vitamin B12; 280 mg d-pantothenic acid; 550 mg of niacin; 9 mg folic acid; 2.0mg d-biotin; 10,000 mg choline; 1800 mg Mn; 2500 mg Fe; 500 mg Cu; 6.0 mg Se; 18,000 mg Lysine; 20,000 mg Methionine; 20.0 mg I; 185.0g of Ca; 80 g of P; 100.0 g of NaCl. No Zn was added in the premix. [2]The number in parentheses indicates the analyzed value.

Supplementary Table 2: Primary antibody information

Gene symbol	Name	Cat. #	Predicted size	Source (Animal)	Company
GAPDH	glyceraldehyde-3-phosphate dehydrogenase	sc-48166	37 kd	Goat (polyclonal)	Santa Cruz Biotechnology, Inc.
Actin	Actin	ab3280	43 kDa	Mouse(monoclonal)	Abcam plc.
γ-H2AX	Anti-gamma H2A.X (phospho S139)	ab26350	16 kDa	Mouse(monoclonal)	Abcam plc.
NF-κB (p65)	Nuclear factor kappa-light-chain-enhancer of activated B cells p65	ab16502	64 kDa	Rabbit (polyclonal)	Abcam plc.
ATM	Ataxia telangiectasia mutated	bs-1370R	370 kDa	Rabbit (polyclonal)	Beijing Biosynthesis Biotechnology CO.
ATR	AT and Rad3-related protein	sc-21848	300 kDa	Goat (polyclonal)	Santa Cruz Biotechnology, Inc.
DNA-PK	DNA dependent protein kinase	bs-2092R	454 kDa	Rabbit (polyclonal)	Beijing Biosynthesis Biotechnology CO.
PP4C	Protein phosphatase 4 catalytic subunit	bs-6428R	35 kDa	Rabbit (polyclonal)	Beijing Biosynthesis Biotechnology CO.
PP6C	Protein phosphatase 6 catalytic subunit	bs-19975	35 kDa	Rabbit (polyclonal)	Beijing Biosynthesis Biotechnology CO.
PP2A	Protein phosphatase 2A	bs-0029R	34 kDa	Rabbit (polyclonal)	Beijing Biosynthesis Biotechnology CO.
PP2ACα	Protein phosphatase2A homologues	bs-4162R	42 kDa	Rabbit (polyclonal)	Beijing Biosynthesis Biotechnology CO.
PP4R2	Protein phosphatase 4 regulator subunit 2	sc-100101	50 kDa	Rabbit (polyclonal)	Santa Cruz Biotechnology, Inc.
PP4R3β	Protein phosphatase 4 regulatory subunit 3 beta	Bs-8328R	97 kDa	Rabbit (polyclonal)	Beijing Biosynthesis Biotechnology CO.
A20	TNFAIP3 (A20)	bs-2803R	90 kDa	Rabbit (polyclonal)	Beijing Biosynthesis Biotechnology CO.
PCNA	Proliferating Cell Nuclear Antigen	bs-2006R	29 kDa	Rabbit (polyclonal)	Beijing Biosynthesis Biotechnology CO.
Cyclin A1	Cyclin A1	bs-5739R	51 kDa	Rabbit (polyclonal)	Beijing Biosynthesis Biotechnology CO.
p53	Tumor protein p53	bs-8687R	53 kDa	Rabbit (polyclonal)	Beijing Biosynthesis Biotechnology CO.
Bax	BCL2-Associated X	bs-4564R	21 kd	Rabbit (polyclonal)	Beijing Biosynthesis Biotechnology CO.
Caspas 8	Caspas 8	bs-0052R	12/55 kd	Rabbit (polyclonal)	Beijing Biosynthesis Biotechnology CO.
Caspas 3	Caspas 3	bs-0081R	28 kd	Rabbit (polyclonal)	Beijing Biosynthesis Biotechnology CO.
Bcl-xl	Bcl-xl	bs-1336R	26 kd	Rabbit (polyclonal)	Beijing Biosynthesis Biotechnology CO.
Bcl-2	Bcl-2	bs-4563R	26 kd	Rabbit (polyclonal)	Beijing Biosynthesis Biotechnology CO.
